# An atlas of dynamic peripheral blood mononuclear cell landscapes in human perioperative anaesthesia/surgery

**DOI:** 10.1002/ctm2.663

**Published:** 2022-01-21

**Authors:** Yang‐Yang Wang, En‐Qiang Chang, Rui‐Lou Zhu, Xiao‐Zhuan Liu, Guang‐Zhi Wang, Ning‐Tao Li, Wei Zhang, Jun Zhou, Xiang‐Dong Wang, Ming‐Yang Sun, Jia‐Qiang Zhang

**Affiliations:** ^1^ Department of Anesthesiology and Perioperative Medicine Center for Clinical Single Cell Biomedicine Henan Provincial People's Hospital, People's Hospital of Zhengzhou University Zhengzhou China; ^2^ Center for Clinical Single Cell Biomedicine Henan Provincial People's Hospital People's Hospital of Zhengzhou University Zhengzhou China; ^3^ Zhongshan Hospital Institute for Clinical Science Shanghai Institute of Clinical Bioinformatics Shanghai Engineering Research for AI Technology for Cardiopulmonary Diseases Fudan University Shanghai China

**Keywords:** anaesthesia, PBMCs, perioperation, scRNA‐seq

## Abstract

**Background:**

The number of patients receiving anaesthesia is increasing, but the impact of general anaesthesia on the patient's immune system remains unclear. The aim of the present study is to investigate dynamics of systemic immune cell responses to anaesthesia during perioperative period at a single‐cell solution.

**Methods:**

The peripheral blood mononuclear cells (PBMCs) and clinical phenomes were harvested and recorded 1 day before anaesthesia and operation, just after anaesthesia (0 h), and 24 and 48 h after anaesthesia. Single‐cell sequencing of PBMCs was performed with 10× genomics. Subsequently, data analysis was performed with R packages: Seurat, clusterProfiler and CellPhoneDB.

**Results:**

We found that the cluster of CD56^+^ NK cells changed at 0 h and the cluster of monocytes increased at 24 and 48 h after anaesthesia. The characteristic genes of CD56^+^ NK cells were mainly enriched in the Jak‐STAT signalling pathway and in cell adhesion molecules (24 h) and carbon metabolism (48 h). The communication between CD14^+^ monocytes and other cells decreased substantially 0 and 48 h after operation. The number of plasma cells enriched in protein export in men was substantially higher than that in women, although the total number in patients decreased 24 h after operation. CD14^+^ monocytes dominated that cell‐cell communications appeared in females, while CD8^+^ NKT cells dominated that cell‐cell communications appeared in male. The number of plasma cells increased substantially in patients with major surgical trauma, with enrichments of pentose phosphate pathway. The communications between plasma cells with other cells varied between surgical severities and anaesthetic forms. The intravenous anaesthesia caused major alterations of cell types, including CD14^+^ monocytes, plasmas cells and MAIT cells, as compared with inhalation anaesthesia.

**Conclusion:**

We initially reported the roles of perioperative anaesthesia/surgery in temporal phenomes of circulating immune cells at a single‐cell solution. Thus, the protection against immune cell changes would benefit the recovery from anaesthesia/surgery.

## Graphical abstract

We initially reported roles of perioperative anesthesia/surgery in temporal phenomes of circulating immune cells at a single cell solution. Thus, the protection against immune cell changes would benefit the recovery from anesthesia/surgery.

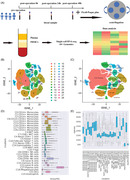

## 1 INTRODUCTION

With the extensive application of single‐cell RNA sequencing (scRNA‐seq) technology, the molecular response of immune cells to various conditions was explored.^1^, ^2^ The anaesthesia accompanied by surgical stress may suppress the immune response and influence the recovery and prognosis through direct action on the immune system or through the activation of the hypothalamic–pituitary–adrenal axis and sympathetic nervous system.^3^ However, the mechanism by which various immune cells are affected by the anaesthesia during the perioperative period remains unclear.

In one study, it was observed that surgery‐induced tissue damage damped the cell‐mediated immune response and increased the infection risk.^4^ Surgical trauma can cause severe immune dysfunction.^5^ The treatment strategy for restoring immune homeostasis after major surgery mainly involves correcting the proinflammatory anti‐inflammatory cell imbalance.^6^The anaesthetics suppress the immunity during the perioperative period^3^ by directly inhibiting the expression and secretion of cellular and neurohumoral immune mediators, thereby the function of immune cells and affecting the inflammatory response. The immune responses to anaesthesia differ depending on the type of anaesthetic.^7^ The effects of inhalation anaesthetics on immunosuppression or immunoactivity depend upon the patient's clinical phenome, the severity of disease and therapies,^8^ although there is still controversy about the effects of inhalation anaesthetics in immune function.^9^

The effects of perioperative anaesthesia/surgery on the temporal phenomes and landscapes of peripheral blood mononuclear cells (PBMCs) at the single‐cell solution remain unclear, even though the perioperative immune response depends on the surgical type. The immune response is related to various relevant factors, such as the anaesthetic used and the underlying disease.^10^ The cellular complexity of PBMCs increases with altered heterogeneity of cell types, different clones and subsets of immune cells. The scRNA‐seq approach can characterize the quantity and functionality of cell types and can provide the details regarding the heterogeneity of the cellular composition. The present study aims to investigate the temporal responses of circulating immune cells to anaesthesia/surgery in the perioperative duration and the potential influencing factors for dynamic landscapes of human PBMCs. The findings will serve as a reference for the development of therapeutic targets and new therapeutic approaches to improve patient outcomes.

## 2 RESULTS

### 2.1 Basic information of patients and peripheral blood samples

**FIGURE 1:**
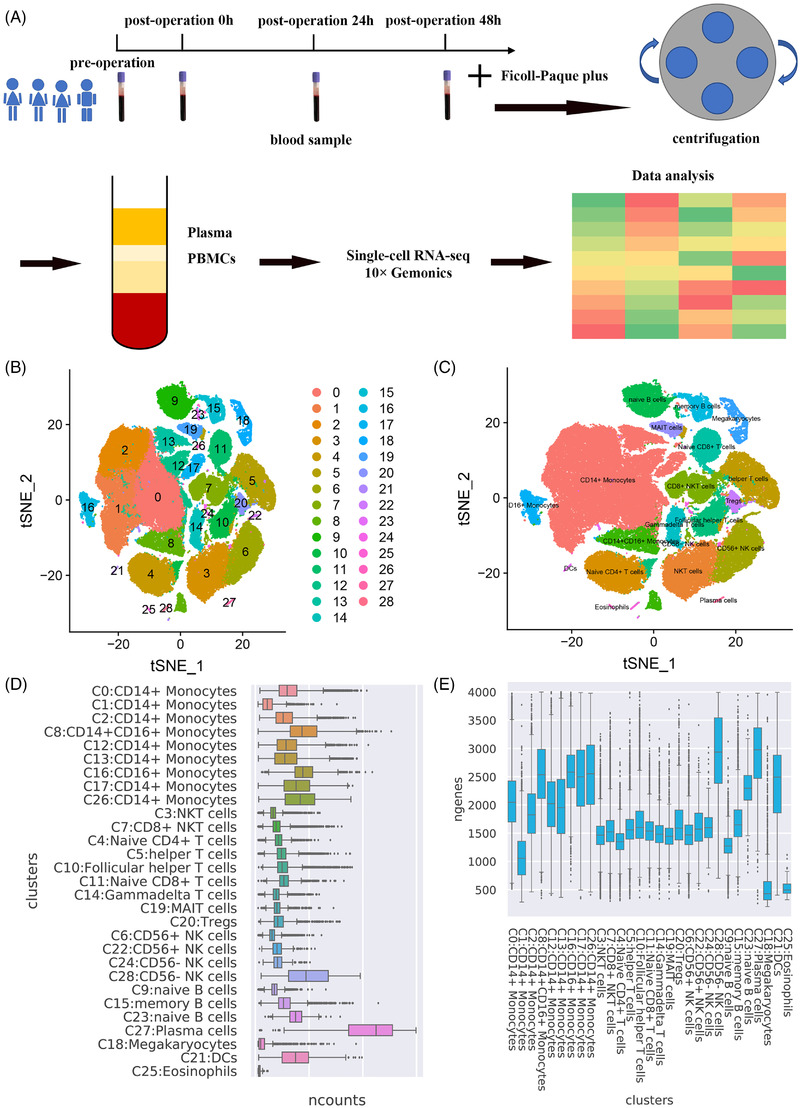
Overview of the PBMCs’ single cells sequence isolated from four patients subjected to general anaesthesia. (A) The flowchart of the experimental process. (B) Cell clusters of all single cells. (C) Cell type of each cluster. (D) The transcript expression abundance of each cluster. (E) The number of genes expressed in all clusters. Abbreviations: DC, dendritic cell; MAIT, mucosal‐associated invariant T

To explore the regulation of immune response by anaesthesia during surgery, we isolated PBMCs of two patients undergoing gynaecological surgery, one patient undergoing total knee arthroplasty (TKA) and one patient undergoing neurosurgery, and performed droplet‐based (10× Genomics Chromium System) scRNA‐seq (Figure 1A). Details on the characteristics of the four patients and their routine blood test results are listed in Table 1. PBMC samples were collected at four time points: pre‐operation,0 h post operation, 24 h post operation and 48 h post operation. The number of PBMCs in each sample and the median number of genes per cell are presented in Table 2. Through scRNA‐seq analysis, these cells were divided into 29 clusters, with each cluster corresponding to a specific type of immune cell. For each cluster, the gene expression abundance and the number of PBMCs differed, while eosinophils (C25) did not belong to PBMCs (Figure 1B‐E).

**TABLE 1:** Demographic characteristics of patients in the study population

Items		1	2	3	4
Age (year)		17	28	63	62
Sex (M/F)		F	F	F	M
Height (cm)		161	163	159	171
Weight (kg)		52	55	62	65
Diagnosis		Ovarian teratoma	Ovarian teratoma	Knee osteoarthritis	Meningioma
Operation		Laparoscopic tumour resection	Laparoscopic tumour resection	Knee replacement	Intracranial tumour resection
Operation time		2 h	2 h	2 h	4 h
Incision type		Minor	Minor	Major	Major
Anaesthetics		Propofol	Sevoflurane	Propofol + Sevoflurane	Propofol + Sevoflurane
WBC (x10^9^/L)	Pro‐	8.04	5.46	4.2	6.6 (65.3%)
	Post‐				9.6
	24			11.79	11.4
	48				8.1
Neutrophils (x10^9^/L)	Pro‐	5.62	2.63	2.1 (49.7%)	4.33 (8.4%)
	Post‐				8.96 (93.4%)
	24			10.56 (89.5%)	10.15 (89.3%)
	48				6.05 (74.7%)
Monocytes (x10^9^/L)	Pro‐	.6	.45	8.2%	8.4%
	Post‐				.6%
	24			.59 (5%)	3.2%
	48				4.5%
Lymphocyte (x10^9^/L)	Pro‐	1.66	2.26	1.62 (38.4%)	1.65 (24.9%)
	Post‐				.57 (5.9%)
	24			.62 (5.3%)	.82 (7.2%)
	48				1.65 (20.3%)
Platelet (x10^9^/L)	Pro‐	294	440	205	178
	Post‐				191
	24			208	126
	48				156
Complications		No	No	No	Ankylosing spondylitis
Prognosis		Good	Good	Good	Good

**TABLE 2:** Estimated cell number and median genes per cell

Sample	Number	Estimated cell number	Median genes per cell
V1	CW7356044	5367	1637
V2	CW7355102	7645	1470
V3	CW7353960	6696	1562
V4	CW7355690	6681	1585
V5	CW7355965	5977	1350
V6	CW7355967	6341	1646
V7	CW7355279	4427	1429
V8	CW7355883	6176	1384
V9	CW7355639	7296	1719
V10	CW7356185	7168	1576
V11	CW7356200	5776	1773
V12	CW7355702	8309	1630
V13	CW5162749	8930	1440
V14	CW5162748	7507	1504
V15	CW7555691	6361	1486
V16	CW5160633	7213	1904

### 2.2 Dynamic characteristics of PBMC clusters in perioperative period

**FIGURE 2:**
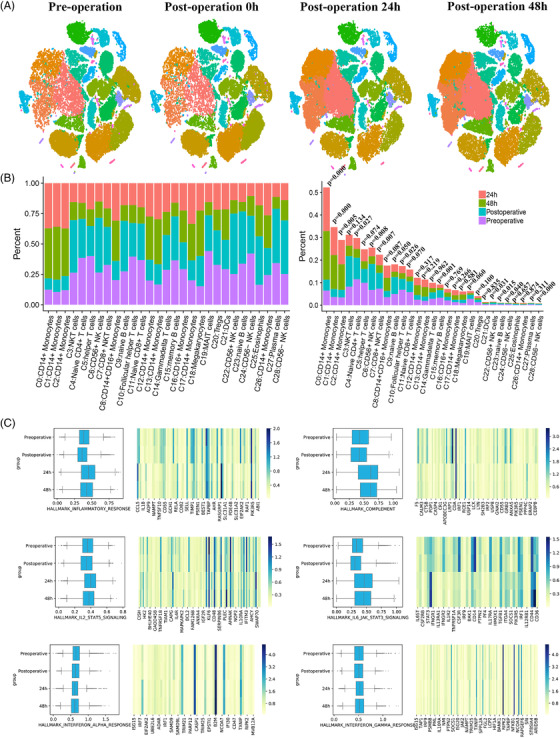
Changes in PBMCs at four time points. (A) Single‐cell t‐SNE diagram at each time point. (B) Percentage change of all clusters at four time points. (C) The overall expression of immune‐related hallmark gene sets at different time points in all samples

At various time points, some clusters exhibited clear changes in their number (Figure 2A). Except eosinophils, which were not eliminated by Ficoll‐Paque Plus, the most notable changes in the cell ratio immediately after surgery were observed in CD56^+^ NK cells (C22), megakaryocytes (C18) and NKT cells (C3), and the changes showed an increasing trend. At 24 h post operation, the cell ratios with the most obvious changes were observed in CD14^+^ monocytes (C1), CD14^+^ monocytes (C2) and CD14^+^ monocytes (C0), which also showed an increasing trend. At 48 h post operation, compared with pre operation stage, the cell ratios that increased the most were still CD14^+^ monocytes (C1), CD14^+^ monocytes (C2) and CD14^+^ monocytes (C0) (Figure 2B). At 0 h post operation, most of the clusters showed a downward trend. Among them, only helper T cells (C5) had a decline rate exceeding 50%. Furthermore, MAIT cells (C19), follicular helper T cells (C10), CD56^–^ NK cells (C24), CD14^+^ monocytes (C0) and CD16^+^ monocytes (C16) decreased by 30‐50%. Eosinophils (C25) were the only cluster to increase by more than 50%. Naïve B cells (C9), NKT cells (C3), megakaryocytes (C18), CD56^+^ NK cells (C6), CD56^–^ NK cells (C28) and CD56^+^ NK cells (C22) increased by 30‐50%, whereas the other clusters increased by less than 30% (Figure S2). Examination of the data of the patients individually revealed crucial information. By the end of the operation, the CD14^+^ monocytes of patient 4 had almost completely disappeared. However, a significant recovery in the number of CD14^+^ monocytes (equivalent to the degree of recovery in the other patients) was made, as indicated in the 24 h post operation data. Similar observations were noted for patient 2, but the reduction in the number of CD14+ monocytes was lower in this patient than in patient 4 (Figure S1).

We performed a hallmark gene set analysis on gene sets at various time points to evaluate immunity‐related changes in hallmark gene set. In the inflammatory response gene sets, the expression of the related genes was inhibited 0 h post operation, and was activated 24 h post operation, after which it declined 48 h post operation. However, gene expression remained higher than that before surgery (Figure 2C). Notably, three gene sets, namely complement, IL2‐STAT5 signalling and IL6‐JAK‐STAT3 signalling, exhibited similar trends. No obvious trend in changes in the two gene sets corresponding to interferon alpha response and interferon gamma response was detected.

**HIGHLIGHT**

- The first study of PBMCs during perioperative period with scRNA sequencing technology
- Dynamic characteristics of PBMC clusters in perioperative period
- By the end of the operation, the CD14^+^ monocytes of certain patient almost completely disappeared

**FIGURE 3:**
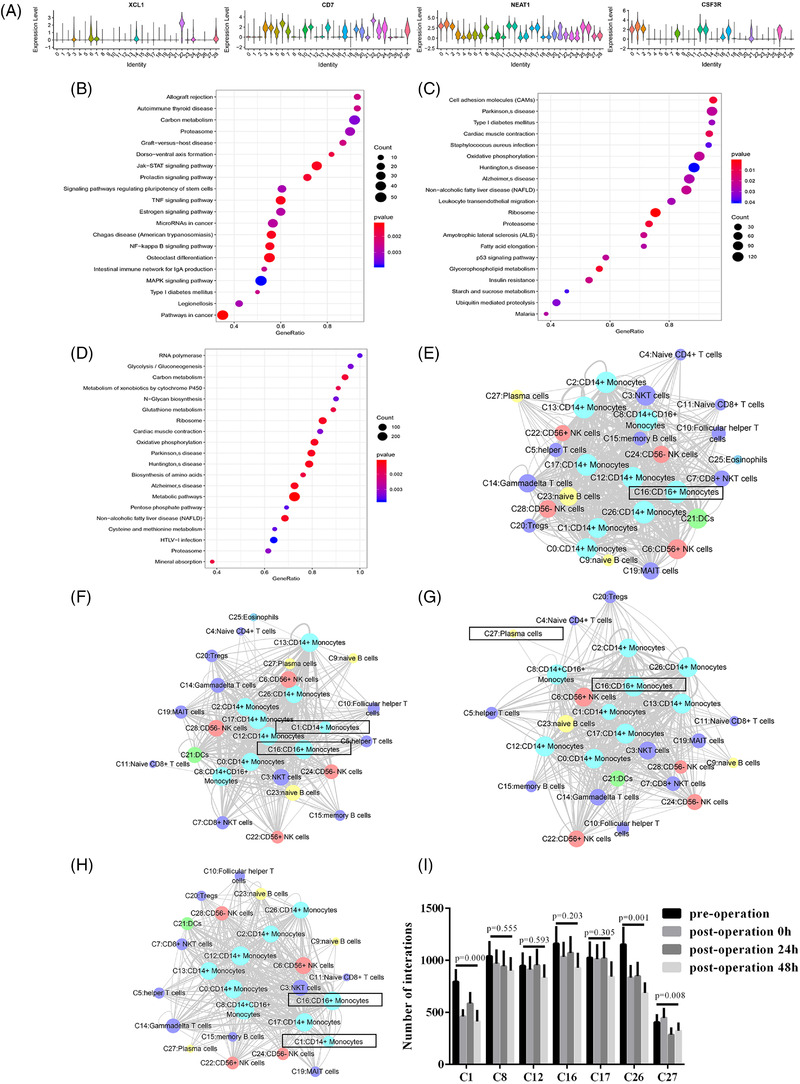
The characteristics of the most variable clusters. (A) The expression of C22 marker genes XCL1 and CD7 and C1 marker genes NEAT1 and CSF3R in all clusters. (B) C22 GSEA analysis results at post‐operation. (C) C1 GSEA analysis result at 24 h post‐operation. (D) C1 GSEA analysis result. (E) Results of cell communication of each cluster before the start of the operation. (F) The cell communication results of each cluster after the operation. (G) Results of cell communication of each cluster 24 h after operation. (H) Results of cell communication of each cluster 48 h after operation

Further analysis was conducted on the clusters CD56^+^ NK cells (C22) and CD14^+^ monocytes (C1), and their numbers changed substantially at various time points. The marker genes of CD56^+^ NK cells (C22) were *XCL1* and *CD7* (Figure 3A), and the marker genes of CD14^+^ monocytes (C1) were *NEAT1* and *CSF3R*. At the three post operation time points, obvious differences were noted in the genes enriched in the GO pathways. These differences were revealed by the gene set enrichment analysis (GSEA) of various clusters (Figures S3‐S5). Compared with the pre operation state, the number of CD56^+^ NK cells (C22) changed the most at the end of surgery. The GSEA of genes expressed in CD56^+^ NK cells (C22) revealed that differential genes were mainly enriched in some functional pathways: carbon metabolism, the JAK‐STAT signalling pathway and so on (Figure 3B). The number of CD14^+^ monocytes (C1) changed the most 24 h post operation, and the main enrichment pathways for the expressed genes involved cell adhesion molecules (CAMs) and leukocyte trans endothelial migration (TEM) (Figure 3C). The most notable change in the cell ratio 48 h post operation was observed in the CD14^+^ monocytes (C1), and the biological pathway of gene enrichment underwent some changes: carbon metabolism replaced CAMs as the main enrichment pathway (Figure 3D).

To function, immune cells rely on signal transmission between cells. This prompted us to explore the interaction between clusters, that is the interaction between cell receptors and ligands. In the perioperative period, the number of cell‐cell communications displayed a downward trend. Both before and immediately after operation, the cluster of CD16^+^ monocytes (C16) interacted the most with other clusters. The interactions of CD14^+^ monocytes (C1) with other clusters changed the most 0 h post operation. CD16^+^ monocytes (C16) interacted most with other clusters 24 h post operation. The most substantial changes relative to baseline were observed in the interactions of the plasma cells (C27). CD16^+^ monocytes (C16) interacted the most with others 48 h post operation. As shown in Figure S6, CD14^+^ monocytes (C1) had the most changes in their interaction with other cells. Analysis revealed that before operation, CD16^+^ monocytes (C16) interacted with other clusters with the most. This cluster also interacted with other clusters with the most in the perioperative period. Following CD16^+^ monocytes (C16), CD14^+^ monocytes (C26) and CD14^+^/CD16^+^ monocytes (C8) showed the most interaction before operation (Figure 3E and Figure S6) and CD14^+^ monocytes (C17) and CD14^+^CD16^+^ monocytes (C8) had the most interactions 0h post operation (Figure 3F and Figure S6). The clusters with the most interactions 24 h post operation were CD14^+^ monocytes (C17) and CD14^+^ monocytes (C12) (Figure 3G and Figure S6). The clusters with the most interactions 48 h post operation were CD14^+^CD16^+^ monocytes (C8) and CD14^+^ monocytes (C17) (Figure 3H and Figure S6). Receptor‐ligand interactions occurred between most clusters, but some interactions were unique to certain clusters. For example, receptor‐ligand pairs involving CD55/ADGRE5, CD74/COPA and CD74/MIF were noted between most clusters. However, receptor‐ligand pairs involving COL26A1/a1b1 complex, SEMA7A/a1b1 complex, COL24A1/a1b1 complex, EPHA2/EFNA4, EFNA4/EPHA4, NRP1/VEGFB, COL18A1/a1b1 complex and CADM1/CADM1 interacted only between certain clusters. At the 0 h post operation, CD14^+^ monocytes (C1) showed the most change in cell communication, showing a significant reduction. At 24 h post operation, the most notable change was observed in plasma cells (C27), which showed a decline in cell‐cell communication. At 48 h post operation, compared with baseline, the most obvious change in clusters was also observed in CD14^+^ monocytes (C1), which showed a decline (Figure 3I).

**FIGURE 4:**
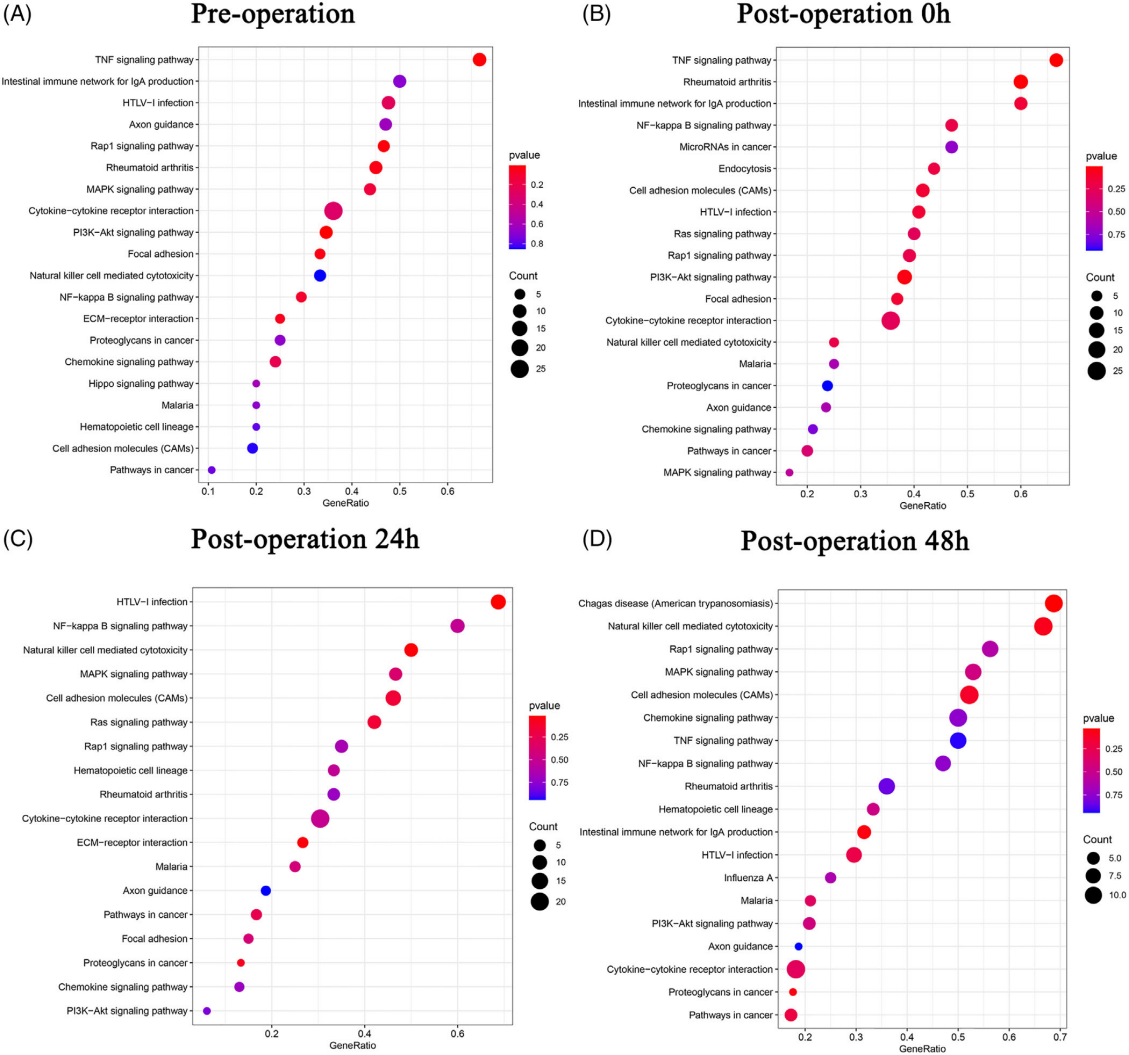
GSEA of receptors and ligands of all clusters analysed by cell–cell communication between different time points. (A) GSEA of receptors and ligands in pre‐operation. (B–D) GSEA of different receptors and ligands at post‐operation 0 h, post‐operation 24 h and post‐operation 48 h compared with pre‐operation

We conducted a GSEA of receptors and ligands that were differentially expressed at various time points and baseline. No significant enrichment pathways (*p* > .25) and few genes (Figure 4) were observed.

### 2.3 Between‐sex difference PBMC cluster characteristics

**FIGURE 5:**
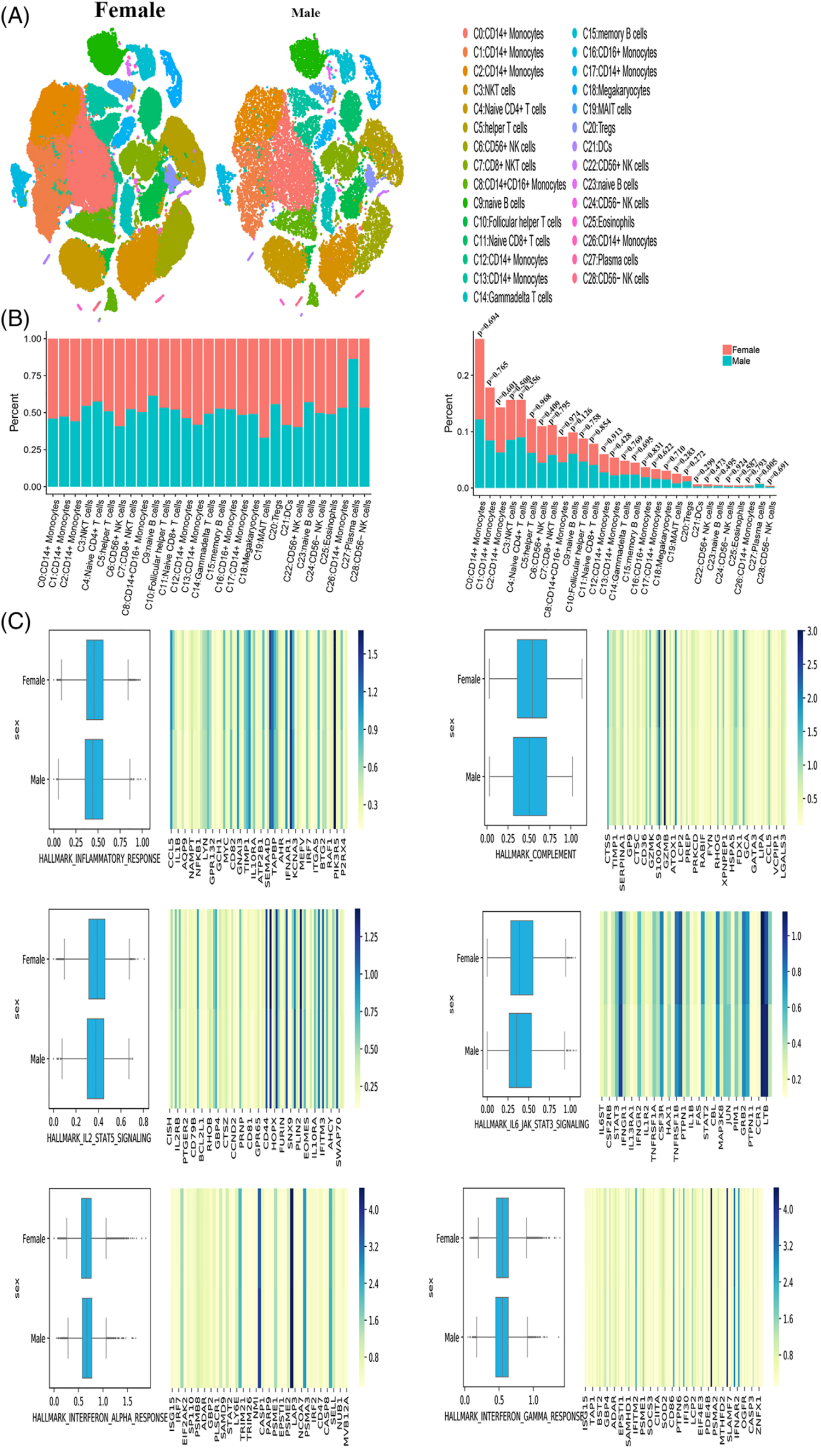
The single cluster of PBMCs in female and male. (A) Single‐cell t‐SNE diagram of female and male. (B) Percentage change of each cluster of different sex. (C) The overall expression of immune‐related hall mark gene sets of two sexes

Some PBMCs displayed clear difference in the cell number changes (Figure 5A). Furthermore, the ratio of clusters was higher in male patients than in female patients, but the number of clusters was almost the same in male and female patients (15 vs. 14). The ratio of MAIT cells (C19) was 50% lower in male patients than in female patients, and CD56^+^ NK cells (C22) and CD56^+^ NK cells (C6) decreased by 30‐50% in males. The ratio of naïve B cells (C9) and plasma cells (C27) was > 1.5 times higher in male patients than in female patients. Notably, plasma cells (C27) increased by 535% in males compared with that in females. Naïve B cells (C23) and helper T cells (C5) increased by 30‐50% in males compared with that in females (Figure S7). Plasma cells (C27) and naïve B cells (C9) exhibited the most obvious changes in terms of the cell ratio (Figure 5B). In the inflammatory response gene sets, a slight suppression of related genes overall was observed in males (Figure 5C). Furthermore, differences were detected in the complement, IL2‐STAT5 signalling and IL6‐JAK‐STAT3 signalling gene sets. However, no clear difference was noted in two gene sets corresponding to the interferon alpha response and the interferon gamma response (Figure 5C).

**FIGURE 6:**
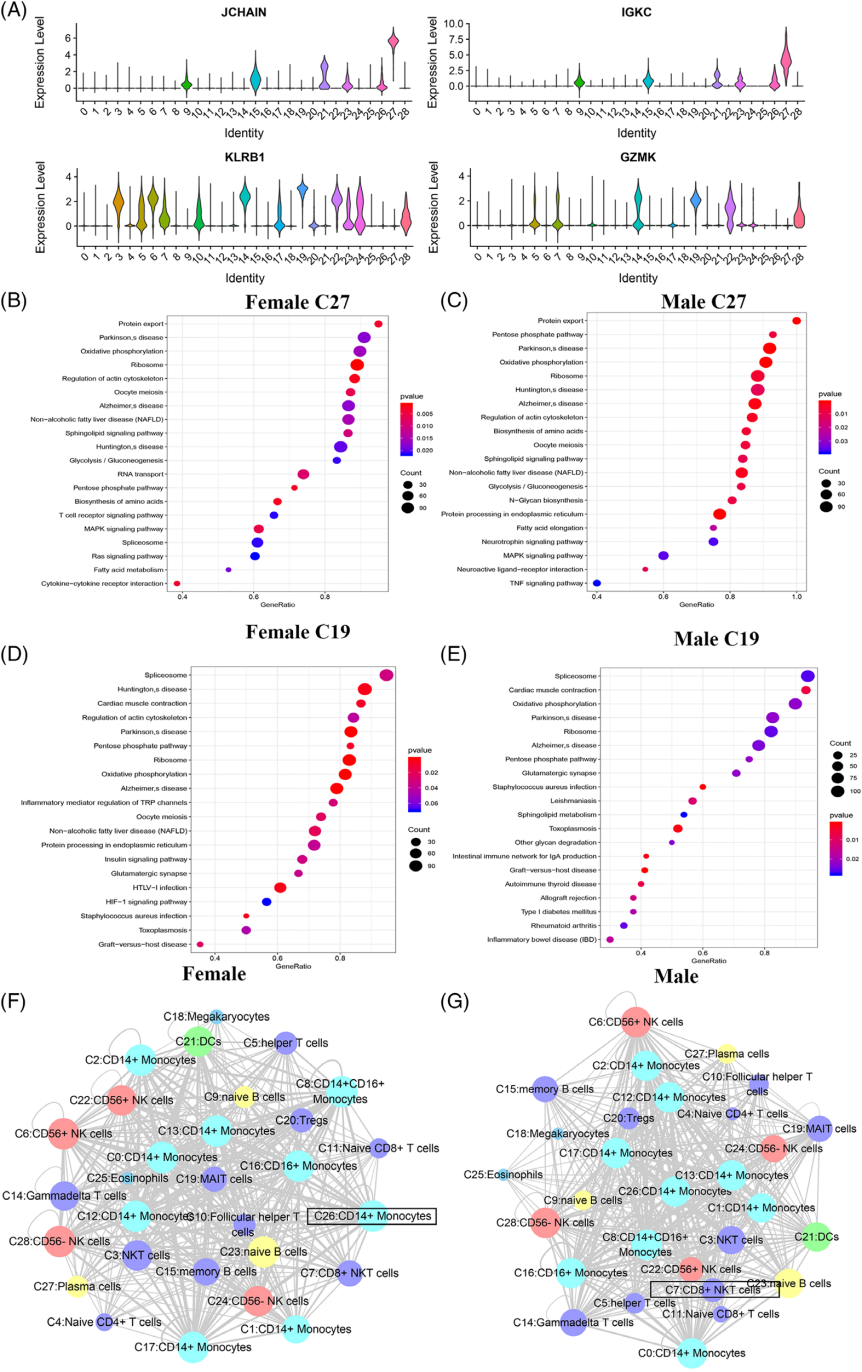
The characteristics of most variable clusters in different sexes. (A) The marker gene JCHAIN, IGKC of C27 and TCL1A, CD79A in C9. (B) C27 GSEA analysis results in female. (C) C27 GSEA analysis results in male. (D) C9 GSEA analysis results in female. (E) C9 GSEA analysis results in female. (F) Results of cell communication of each cluster in female. (G) Results of cell communication of each cluster in male

Plasma cells (C27) and naïve B cells (C9), which displayed the most substantial changes both in the male patients and female patients, were subjected to further analysis. The marker genes of plasma cells (C27) were *JCHAIN* and *IGKC* (Figure 6A), and the marker genes of naïve B cells (C9) were *TCL1A* and *CD79A*. Notable sex‐specific differences were found in the genes enriched in the GO pathways, as revealed by the GSEA (Figures S8 and S9). The GSEA of the genes expressed by plasma cells (C27) in women was compared with the average level, and the differential genes were mainly enriched in some functional pathways: protein export and oxidative phosphorylation (Figure 6B). Furthermore, the GSEA of plasma cells (C27) in males revealed similar enrichment results, while some of the differential genes were enriched in the MAPK signalling pathway in males (Figure 6C). In females, naïve B cells (C9) expressed genes were mainly enriched in pathways, including glyoxylate and dicarboxylate metabolism, and the cGMP‐PKG signalling pathway (Figure 6D). In males, naïve B cells (C9) differential genes were enriched in platelet activation, biosynthesis of amino acid and Fc gamma R‐mediated phagocytosis (Figure 6E).

The interaction between the receptors and ligands differed between sexes (Figure 6G, H). In females, CD14^+^ monocytes (C26) interacted the most with other clusters. The corresponding cluster in males was CD8^+^ NKT cells (C7). The cluster with the largest between‐sex difference was CD8^+^ NKT cells (C7) (Figure S10). Some receptor‐ligand interactions occurred between most clusters; some interactions were unique to certain clusters. In females, for example SELL/SELPLG, CD74/MIF and MIF/TNFRSF14 interacted with most clusters. However, CADM1/CADM1, EPHA4/EFNB1, EPHB1/EFNB1 and NRP1/VEGFB interactions were found only between specific clusters in females. It displayed slight variations in terms of specifically expressed ligand‐receptor pairs in males: NRP1/VEGFB, COL18A1/a1b1 complex, COL4A4/a1b1 complex, CADM1/CADM1, CCR3/CCL28, EPHA1/EFNA4, EPHB1/EFNB1, SEMA7A/a1b1 complex, COL26A1/a1b1 complex, NRP1/VEGFA and EPHA2/EFNA4.

**FIGURE 7:**
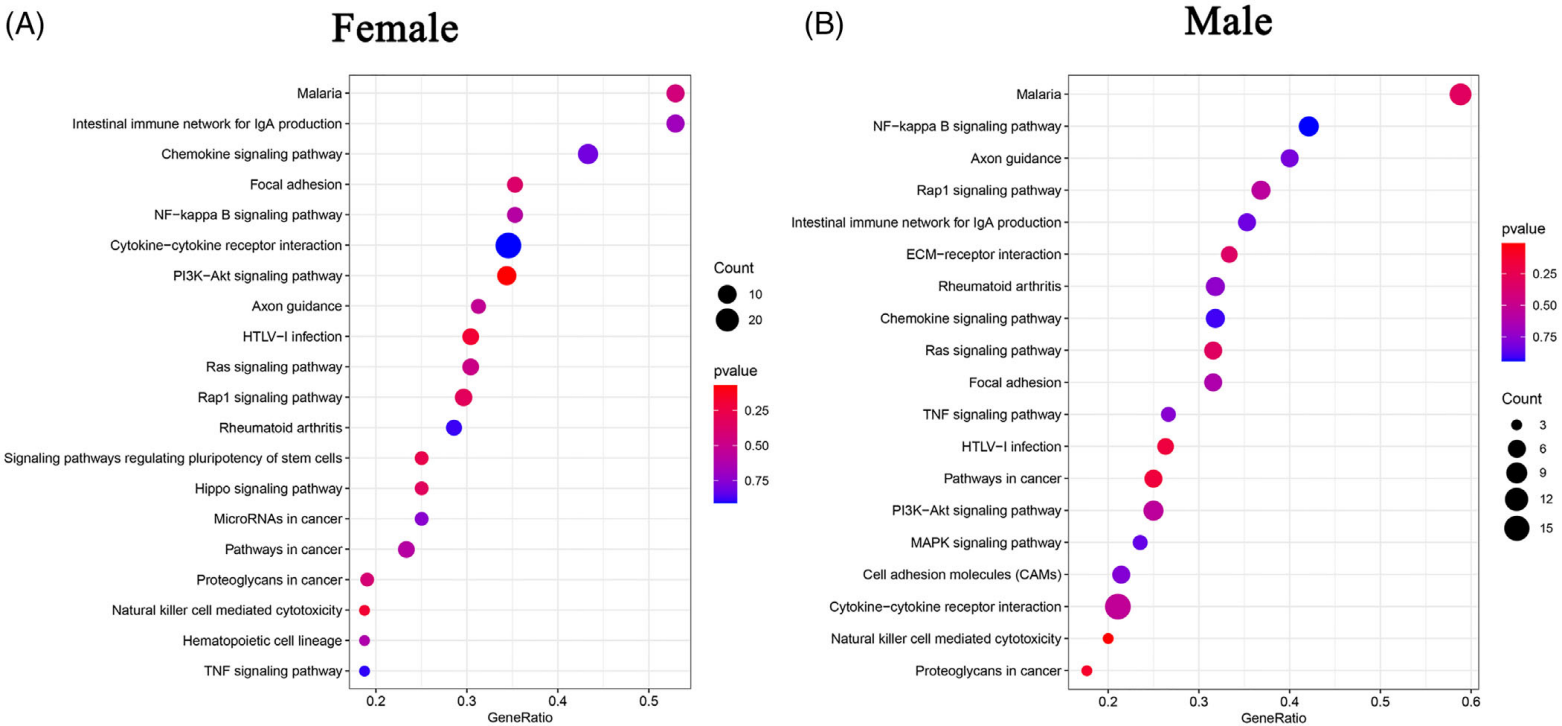
GSEA of receptors and ligands of all clusters analysed by cell–cell communication between different genders

GSEA revealed no significant differences in the receptor–ligand pairs differentially expressed between sexes (*p* > .05) (Figure 7).

### 2.4 Influence of surgical trauma on PBMC cluster characteristics

**FIGURE 8:**
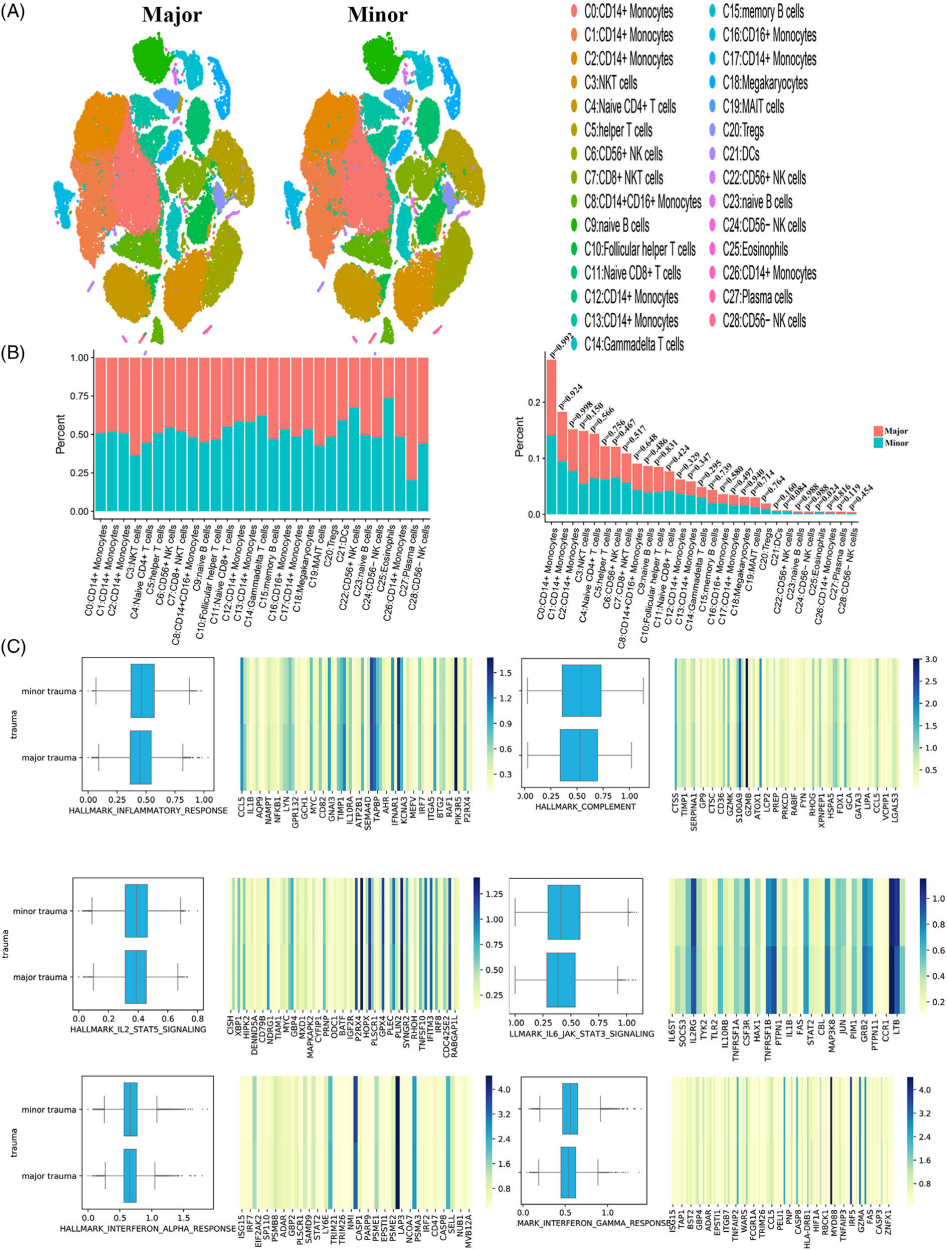
The single cluster of PBMCs in variable surgeries. (A) Single‐cell t‐SNE diagram of major and minor trauma. (B) Percentage change of each cluster of major and minor trauma. (C) The overall expression of immune‐related hall mark gene sets of major and minor trauma

The effects of surgical trauma on PBMCs may vary with type of surgery and the level of trauma sustained. The four patients underwent gynaecological surgery (hysteroscopic surgery and minor trauma), TKA (major trauma) and neurosurgery (major trauma). Comparing the two levels of trauma, some of the patients’ PMBC clusters showed obvious changes in cell number (Figure 8A). Because hysteroscopic surgery has the smallest effect on patients of all the surgeries considered, it was regarded as a reference. Ratio of certain clusters in patients with major trauma decreased significantly. Eosinophils (C25) and CD56^+^ NK cells (C22) decreased more than 50%, and γδ T cells (C14) and dendritic cells (DCs) (C21) decreased by 30‐50%. The ratios of plasma cells (C27) increased most significantly (by 292%) in patients with major surgical trauma. MAIT cells (C19) and NKT cells (C3) increased by 30‐50% (Figure 8B and Figure S11). In the inflammatory response gene sets, no significant differences were found in patients with minor and major surgical trauma (Figure 8C). And similar observations were made in the four gene sets, namely complement, IL2‐STAT5 signalling, interferon alpha response and interferon gamma response but in the IL6‐JAK‐STAT3 signalling gene set.

**FIGURE 9:**
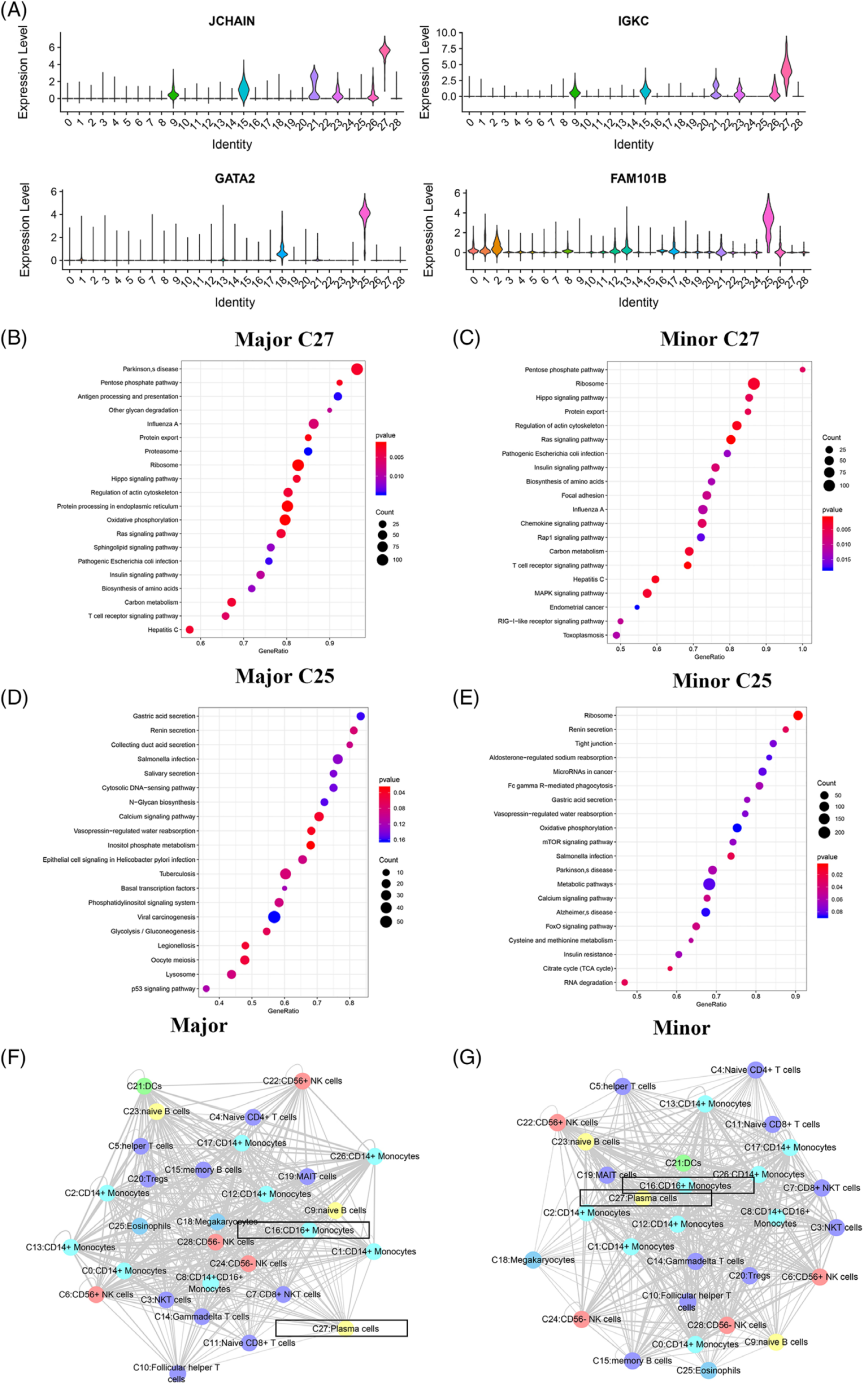
The characteristics of most variable clusters in different surgeries. (A) The marker gene KLRB1, GZMK of C27 and GATA2 and FAM101B in C25. (B) C27 GSEA analysis results in major trauma. (C) C27 GSEA analysis results in minor trauma. (D) C25 GSEA analysis results in major trauma. (E) C25 GSEA analysis results in minor trauma. (F) Results of cell communication of each cluster in major trauma. (G) Results of cell communication of each cluster in minor trauma

Further analysis was conducted on plasma cells (C27) and eosinophils (C25), because they displayed the most changes at the two trauma levels. The marker genes of plasma cells (C27) were *JCHAIN* and *IGKC* (Figure 9A), and the marker genes of eosinophils (C25) were *GATA2* and *FAM101B*. Regarding the two levels of surgical trauma considered, obvious differences were detected in the genes enriched in the GO pathways, as determined by the GSEA of clusters (Figures S12 and S13). The GSEA of genes expressed by plasma cells (C27) in patients with major trauma demonstrated that differential genes were mainly enriched in some GO pathways: pentose phosphate pathway, Ribosome and Hippo signalling pathway (Figure 9B). The GSEA of plasma cells (C27) in patients with minor trauma revealed different enrichment results compared with that in patients with major trauma (Figure 9C). The GSEA of genes expressed by eosinophils (C25) in patients with major trauma showed some GO pathways: gastric acid secretion and p53 signalling pathway (Figure 9D), while the GSEA of eosinophils (C25) in patients with minor trauma showed different enrichment results: differential genes were mainly enriched in the mTOR signalling pathway and calcium signalling pathway (Figure 9E).

Moreover, the receptor‐ligand interactions differed according to the surgical trauma level (Figure 9F, G). In minor surgeries, the cluster that interacts most with other cells was CD16^+^ monocytes (C16), which was the same in major surgeries. The cluster exhibiting the largest difference in cell‐cell communication between the two groups was plasma cells (C27) (Figure S14). Analysis of the effects of surgical trauma levels on cell‐cell communication revealed that no ligand‐receptor pair existed independently between two clusters, and most of the communication patterns were similar among the clusters.

**FIGURE 10:**
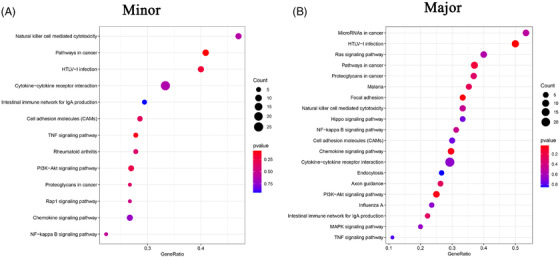
GSEA of receptors and ligands of all clusters analysed by cell–cell communication between different trauma

Next, we performed a GSEA of receptors and ligands that were differentially expressed between the patients undergoing major versus minor surgery. No significant signalling pathway was found (*p* > .05) (Figure 10).

### 2.5 Effects of different general anaesthetics on PBMC characteristics

**FIGURE 11:**
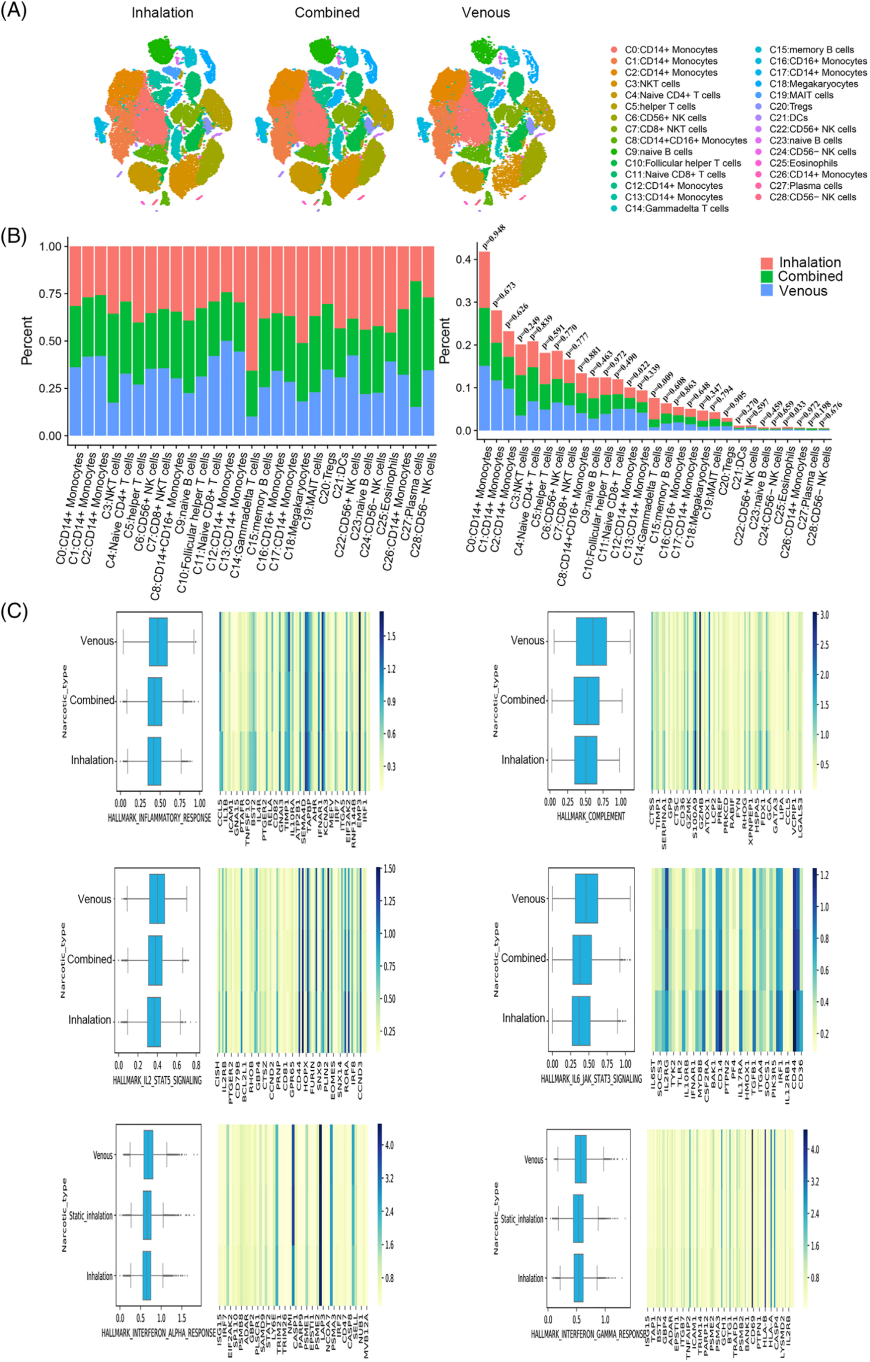
The single cluster of PBMCs in variable anaesthetics. (A) Single‐cell t‐SNE diagram of different anaesthetics. (B) Percentage change of each cluster of different anaesthetics. (C) The overall expression of immune‐related hall mark gene sets of different anaesthetics

Sevoflurane and propofol are the drugs most commonly employed for maintaining general anaesthesia in clinical practice. Some anaesthesiologists tend to use these drugs independently, whereas others use the two in combination to reduce the risk of side effects. Notably, the patient's condition affects anaesthesiologists' decision making. Therefore, we analysed PBMC characteristics on the basis of the anaesthetics administered. Overall, PBMCs exhibited slight differences in some clusters in patients receiving inhalation anaesthesia (1), intravenous (1) or combined anaesthesia (2) (Figure 11A). The general decline in the ratio of PMBC clusters was more in patients receiving combined anaesthesia than in that receiving inhalation anaesthesia. The ratio of eosinophils (C25) and γδ T cells (C14) changed by more than 50%, and CD56^+^ NK cells (C22), DCs (C21), and megakaryocytes (C18) decreased by 30‐50%. Regarding changes in ratios, the largest increase was observed in plasma cells (C27) (by 262%). The ratio of naïve CD4^+^ T cells (C4), NKT cells (C3) and CD56^–^ NK cells (C28) increased by 30‐50%. Compared with patients receiving inhalation anaesthesia, patients receiving intravenous anaesthesia had a larger decline in clusters relative to base level. A reduction of more than 50% was noted in γδ T cells (C14), megakaryocytes (C18), NKT cells (C3) and naïve B cells (C23). Regarding CD56^–^ NK cells (C24), naïve B cells (C9), MAIT cells (C19), helper T cells (C5) and memory B cells (C15), a decline of 30‐50% was documented. The other clusters increased in ratio, with the largest increase of 107% observed in CD14^+^ monocytes (C12). CD14^+^ monocytes (C13), CD14^+^ monocytes (C1) and CD14^+^ monocytes (C2) increased by 30‐50% (Figure S15). Compared with that in patient receiving simple inhalation anaesthesia, in patients receiving total intravenous anaesthesia, the cluster with the most obvious change in cell ratio was CD14^+^ monocytes (C12); specifically, they underwent a significant increase. Compared with that in patient receiving simple inhalation anaesthesia, the largest change in the PBMCs ratio was observed in plasma cells (C27) in patients receiving combined anaesthesia (Figure 11B).

In hallmark gene set analysis, we focused on the six gene sets related to the immune response (Figure 11C). In general, relative to total intravenous anaesthesia, the effect of combined and inhalation anaesthesia on immune system‐related gene sets was mainly inhibitory. Compared with total intravenous anaesthesia, combined and inhalation anaesthesia significantly inhibited gene expression in the inflammatory response gene set. Similar findings were obtained for the five gene sets, namely complement, IL2‐STAT5 signalling, IL6‐JAK‐STAT3 signalling, interferon alpha response and interferon gamma response. The most obvious difference was found in the IL6‐JAK‐STAT3 signalling pathway.

**FIGURE 12:**
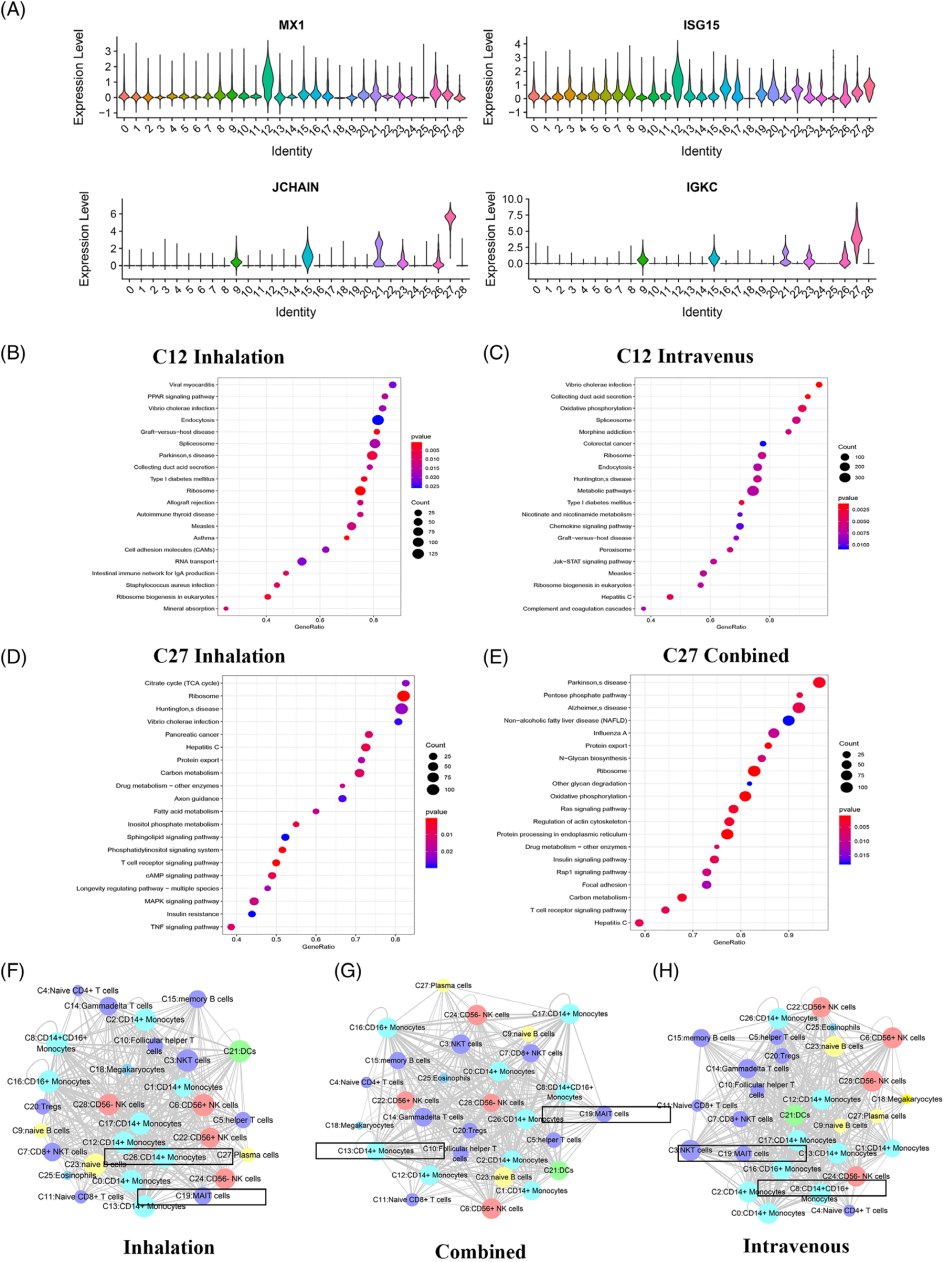
The characteristics of most variable clusters in different anaesthetics. (A) The marker gene LTB, LDHB of C12 and JACHAIN and IGKC in C27. (B) C12 GSEA analysis results in patients with inhalation anaesthesia. (C) C12 GSEA analysis results in patients with intravenous anaesthetics. (D) C27 GSEA analysis results in patients with inhalation anaesthesia. (E) C27 GSEA analysis results in patients with combined anaesthetics. (F) Results of cell communication of each cluster in inhalation anaesthesia. (G) Results of cell communication of each cluster in combined anaesthesia. (H) Results of cell communication of each cluster in intravenous anaesthesia

For CD14^+^ monocytes (C12) and plasma cells (C27), the number of which varied the most with the route of anaesthesia was subjected to further analysis. The marker genes of plasma cells (C27) were *JCHAIN* and *IGKC*, and those of CD14^+^ monocytes (C12) cluster were *MX1* and *ISG15* (Figure 12A). For different anaesthetics, obvious differences were found in the genes enriched in the GO pathways, as determined by the GSEA of clusters (Figures S16‐S18). The GSEA of genes expressed by CD14^+^ monocytes (C12) in patients receiving inhalation anaesthesia demonstrated differential genes was mainly enriched in certain functional pathways: oxidative phosphorylation, PPAR signalling pathway and CAMs (Figure 12B). Similar enrichment results were noted in the GSEA of genes expressed by CD14^+^ monocytes (C12) in patients receiving total intravenous anaesthesia. Moreover, some differential genes were enriched in oxidative phosphorylation, morphine addiction and JAK‐STAT signalling pathway (Figure 12C). In patients receiving inhalation anaesthesia, genes expressed by plasma cells (C27) were mainly enriched in pentose phosphate pathway, cAMP signalling pathway, MAPK signalling pathway and TNF signalling pathway (Figure 12D). In patients receiving combined anaesthesia, the differential genes expressed by plasma cells (C27) were enriched in pentose phosphate pathway, Ras signalling pathway, Rap1 signalling pathway and focal adhesion (Figure 12E).

Receptor‐ligand interactions varied with the route of anaesthesia (Figure 12G, H). Comparing the three anaesthetic routes with regard to their effects on immune cell communication, the interaction between CD14^+^ monocytes (C26) and other clusters was the highest in patients receiving inhalation anaesthesia. In patients receiving combined anaesthesia, CD14^+^ monocytes (C13) had the most interactions with other clusters. A greater change in MAIT cells (C19) in the number of interactions was significantly fewer than that in patients receiving inhalation anaesthesia, with an overall increasing trend. The most interactions between CD14^+^CD16^+^ monocytes (C8) and other clusters were noted in patients receiving intravenous anaesthesia. MAIT cells (C19) in patients receiving intravenous anaesthesia displayed more changes than those in patients receiving inhalation anaesthesia (Figure S19). Analysis revealed that in patients receiving inhalation, combined and intravenous anaesthesia, CD14^+^ monocytes (C26), CD14^+^ monocytes (C13) and CD14^+^CD16^+^ monocytes (C8) interacted the most with other cell clusters. Receptor‐ligand interactions occurred between most clusters, but some interactions were unique to certain clusters. For example, in patients receiving inhalation anaesthesia, SELL/SELPLG, CD74/MIF and MIF/TNFRSF14 interactions were noted in most clusters. Interactions of NRP1/VEGFB, CADM1/CADM1, EFNA4/EPHA4, EPHA2/EFNA4, CD70/TNFRSF17 and EPHA1/EFNA4 receptor‐l were found only between specific clusters in patients receiving inhalation anaesthesia. Furthermore, CD74/COPA, CD74/MIF and MIF/TNFRSF14 receptor‐ligand pairs interacted with most clusters in patients receiving combined anaesthesia. By contrast, EPHB1/EFNB1, NRP1/VEGFB, CADM1/CADM1 and CCR3/CCL28 receptor‐ligand pairs interacted only between certain clusters in patients receiving combined anaesthesia. In patients receiving intravenous anaesthesia, CD74/COPA, CD74/MIF and MIF/TNFRSF14 receptor‐ligand pairs interacted between most cell subpopulations in patients receiving intravenous anaesthesia, whereas FLT4/PDGFC, NRP1/VEGFA, CCR3/CCL28, SELP/CD34, EPHB1/EFNB1 and CADM1/CADM1 receptor‐ligand pairs interacted only between certain clusters in patients receiving intravenous anaesthesia.

**FIGURE 13:**
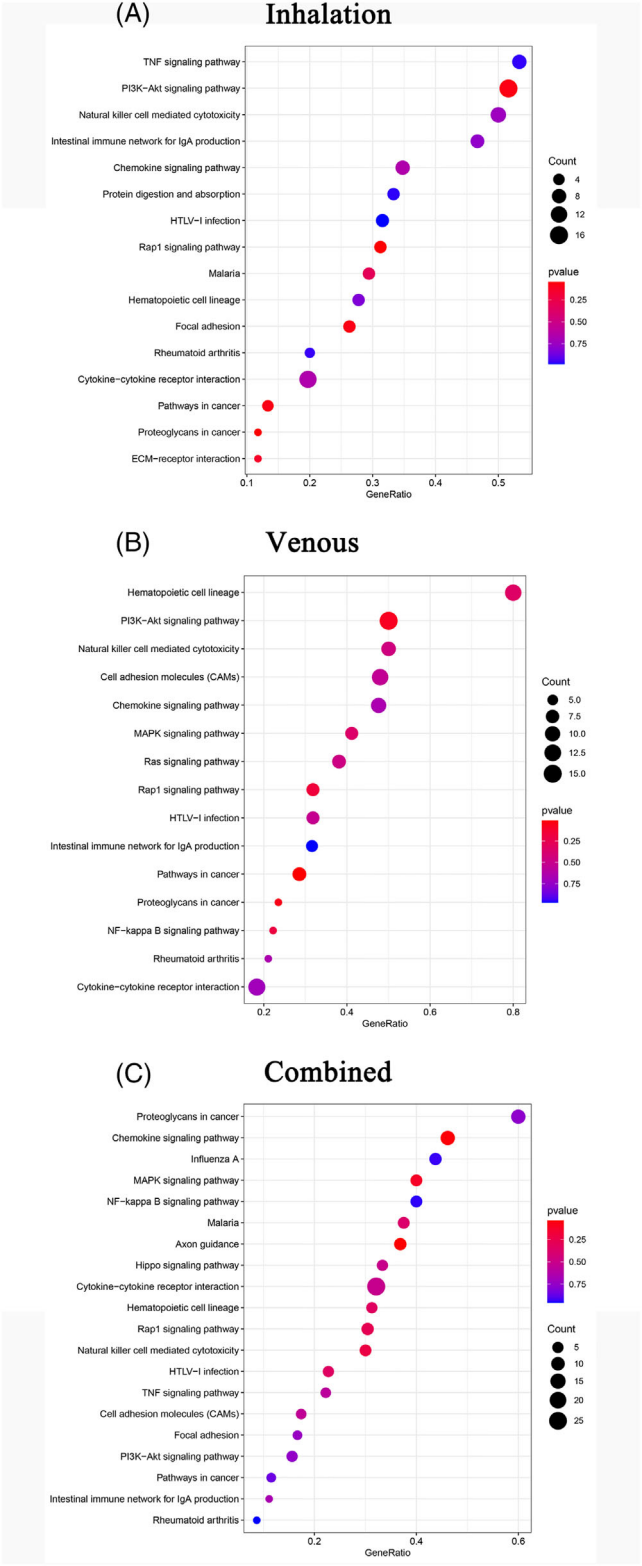
GSEA of receptors and ligands of all clusters analysed by cell–cell communication between different anaesthetics. (A) GSEA of receptors and ligands with venous anaesthesia. (B) GSEA of receptors and ligands with combined anaesthesia. (C) GSEA of different receptors and ligands with inhalation anaesthesia

We conducted a GSEA of receptors and ligands that were differentially expressed according to the route of anaesthesia. No significant signalling pathway was found (*p* > .05) (Figure 13).

## 3 DISCUSSION

In this study, we collected blood samples from four representative patients at four time points in the perioperative period and extracted PBMCs from the samples, conducted scRNA‐seq and analysed the characteristic changes in PBMCs. This preliminarily study demonstrated an atlas of dynamic PBMC landscapes in human perioperative period.

In contrast to most studies on anaesthesia, which tend to focus on specific diseases, this investigation centred on the entire overall process of general anaesthesia. We determined the common dynamic changes in the immune system caused by various routes of anaesthesia. We collected samples at multiple time points and examined the changes in gene expression at each of these points relative to baseline. Genes with no dynamic features were ignored. The change characteristics of each cluster at dynamic time points are the product of the effects of anaesthesia and surgical factors.

In certain patients, anaesthesia/surgery may cause the temporary disappearance of CD14+ monocyte. Herein, CD14^+^ monocyte accounted for a high ratio of all cells for patients 1–3. In patient 4, this cell cluster disappeared after anaesthesia, which is one of our major findings. The most notable baseline characteristic of patient 4 is that he had ankylosing spondylitis. His CD14^+^ monocyte almost disappeared at the end of the operation, but 24 h post operation, the cluster had more than recovered, exceeding the number from pre operation. Moreover, a high ratio of CD14^+^ monocyte was maintained until at least 48 h post operation. These results may be related to abnormalities of the patients’ immune system. The sensitivity of CD14^+^ monocyte to anaesthesia is elevated in patients with abnormal immune system. Pathology testing of the postoperative tumour of patient 2 with a large CD14^+^ monocyte reduction revealed that the tumour was a teratoma, which can cause autoimmune encephalitis. Overall, the results suggest that the process of anaesthesia/surgery shows a higher impact on the CD14^+^ monocytes of patients with autoimmune diseases.

Other cell clusters that underwent changes were also determined to have strong dynamic evolution characteristics. CD56^+^ NK (C22) exhibited strong dynamic features and the related study has demonstrated that general anaesthesia can inhibit the immune function of NK cells 24 h after breast cancer surgery. Compared with sevoflurane‐opioid anaesthesia, the propofol‐paraspinal nerve block anaesthesia has a stronger inhibitory effect on NK cells.^11^ By coculturing NK cells with propofol, the study has revealed that propofol can enhance the function of NK cells in patients with oesophageal cancer.^12^ Herein, the genes expressed by CD56^+^ NK (C22) cells were clearly enriched in the JAK‐STAT signalling pathway. Evidence indicates that the JAK‐STAT pathway is involved in sensory cytokine formation, and that treatments targeting the JAK‐STAT signalling cascade molecules are effective for pain relief.^13^ In the present study, the ratio of CD14^+^ monocytes (C1) was maintained at a high level. Study has shown that monocytes increase significantly 24 h post operation and participate in postoperative delirium.^14^ As CD14^+^ monocytes (C1) increase in ratio, they may function through the biological processes of leukocyte TEM and CAMs. CAMs are members of the immunoglobulin family and are regulated by inflammatory interleukins.^15^ CAMs can promote wound healing, and ICAM‐1 promotes the recruitment and adhesion of leukocytes to the activated endothelium by binding to integrins, thereby exerting its effect and promoting migration through endothelial cells.^16^ Most importantly, surgery would influence CAMs.^17^ The effect of anaesthetics on the carbon metabolism of various organs has been confirmed,^18^, ^19^ but systematic research on the corresponding effect on immune cell carbon metabolism is lacking. In the present study, irrespective of the perioperative period, monocytes engaged in the most extensive cell‐cell communication of all clusters examined. However, at 24 h post operation, the cluster with the most changes in cell‐cell communication switched to plasma cells (C27). The role played by unique ligand receptors on certain immune cells under anaesthesia remains unclear. Further experiments are warranted to determine the functions of these receptors and to thereby enrich the understanding of cell‐cell communication in the process of surgical anaesthesia.

Over the perioperative period, high similarity of PBMCs dynamic changes was observed between male and female patients. Study has demonstrated that the immune system of men and women differs substantially.^20^ Sex hormones play an essential role in both innate immunity and adaptive immunity, leading to considerable differences in various immune processes, such as those involving trauma and infection.^21^ However, no significant difference was noted between male and female patients. Considering our small sample size and the fact that there were three female patients and one male patient, no reliable conclusions can be drawn from these results.

Surgical trauma can affect the dynamic changes occurring in PBMCs during the perioperative period. Specifically, surgical trauma activates various stress responses, causing the release of catecholamines, cortisol and inflammatory mediators,^22^ which further strengthens the perioperative inflammatory response and immune suppression,^23^, ^24^ and even leads to immune cell dysfunction, thus triggering changes in PBMCs.^25^ Effector B cells may have been the cluster the most affected by surgical trauma in the present study. Moreover, IL6‐JAK‐STAT3 signalling gene set may be related to the surgical trauma degree; this observation is consistent with that in another investigation.^26^ It was reported that PI3K‐Akt signalling pathway plays a crucial role in the processes of bleeding and surgical trauma.^27^ The pentose phosphate pathway is a pivotal bypass of intracellular carbon metabolism. It also affects other metabolic processes,^28^ producing antioxidants that protect cells from oxidative damage.^29^ Long‐term exposure to sevoflurane converts the pentose phosphate pathway into the glycolytic pathway.^30^ In addition to surgical trauma, age exerted effects on the patients’ immune cells. The two patients with minor surgical trauma were young, and the two patients with major trauma were significantly older than these two young people. Age is a vital factor that affects immunity.^31^ The impact of surgical trauma on immune cells is emphasized in this paper because we believe that it is greater than that of age.

The dynamic changes in PBMCs during the perioperative period differed according to the route of anaesthesia. The effect of different anaesthetics on immunity has been the focus of substantial body of anaesthesiology research.^32^, ^33^ Studies have been conducted on patients’ immune status.^34^, ^35^, ^36^ Other investigation has explored whether anaesthesia can inhibit the immune response of patients with cancer, thereby affecting their long‐term outcomes.^37^, ^38^ Studies have also examined the effects of anaesthetics on the number and apoptosis of immune cells, and some studies have examined the changes in inflammatory factors and oxidative stress levels. However, in‐depth research on the mechanisms by which anaesthesia drives changes in immune cells is lacking, and the evidence on this topic is inconsistent. Studies have confirmed that anaesthetics can affect monocytes, and that local anaesthetics can inhibit monocyte function.^39^ However, few studies have been conducted on the interaction of plasma cells and anaesthetics. In the present study, the pathways most affected by intravenous general anaesthesia were oxidative phosphorylation and the JAK‐STAT signalling pathway. In an animal model, compared with propofol anesthetized rats, isoflurane anesthetized rats exhibited higher oxidative stress, decreased oxidative phosphorylation protein expression and electron transport chain activity, and increased organ protective protein expression.^40^ Another study reported that propofol administration affected the JAK‐STAT signalling pathway.^41^ Other investigations have indicated that propofol affects this molecular pathway in nerve cells^42^ and tumour cells^43^ among other cell types. Only intravenous general anaesthesia had a different effect and involved naïve CD8^+^ T cells.

In sum, anaesthesia/surgery exerted effects on the temporal phenomes of circulating immune cells at a single‐cell solution. Protection against such immune cell changes would benefit recovery from anaesthesia/surgery.

## 4 METHODS

The study protocol was approved by the Ethics Review Committee of Henan Provincial People's Hospital (2019‐44). All patients provided written informed consent regarding the use of their blood samples for research purpose.

### 4.1 Patient information and analytical strategies

Table 1 presents the characteristics of the patients, including their basic information, surgery type, and anaesthetics used during the anaesthesia process, as well as the results of routine blood tests conducted at each time point. To study changes in PBMCs from patients during the perioperative period, peripheral blood samples were collected at four time points, namely before anaesthetic administration (pre operation), immediately after the end of the operation (post operation 0 h), 24 h after the operation (post operation 24 h) and 48 h after the operation (post operation 48 h). In subsequent analysis, various analysis strategies were employed to determine the dynamic changes. First, the data of all four patients were combined, and PBMC changes at different time points were analysed. In the second comparison method, sex‐related factors were considered and compared between one male patient and three female patients. Third, we analysed the effects of neurosurgery (one patient), gynaecological surgery (two patients) and TKA (one patient) on differences in PBMC clusters. In the fourth comparison, we analysed differences in PBMCs across inhalation anaesthesia (one patient), combination of intravenous and inhalation anaesthesia (two patients) and intravenous anaesthesia (one patient). None of the patients received immune‐related treatment from the pre operation to 48 h post operation.

### 4.2 Peripheral blood collection and mononuclear cell separation

At fixed time points, patients’ blood was collected by a dedicated person. Blood collected from each patient (4 ml) was temporarily stored in a blood collection tube containing ethylenediaminetetraacetic acid and was placed in an ice box for transport. Ficoll‐Paque Plus was used to extract PBMCs: 3 ml of Ficoll‐Paque Plus reagent was added to a 10‐ml centrifuge tube; then, the whole blood was slowly poured into the centrifuge tube to suspend the blood above the reagent; this entire process was executed on a sterile operating table. Then, centrifugation at 400 *g* at 18℃ was performed for 30 min. Subsequently, a sterile pipette was used to draw out the upper layer of the sample containing plasma and platelets, and another sterile pipette was used to transfer mononuclear cells into a sterile centrifuge tube. Furthermore, 6 ml of balanced salt solution was added to the monocytes in the centrifuge tube, and the cells were suspended gently and then centrifuged at 400 *g* for 10 min at 18℃. After the supernatant was removed, 6 ml of balanced salt solution was added to resuspend cells, and the cells were centrifuged again at 400 *g* for 10 min at 18°C to obtain mononuclear cells. Finally, the supernatant was discarded, and the cells were resuspended in 1 ml of complete medium.

### 4.3 Preparation of 10× genomics library and RNA‐sequencing

Based on the 10× Genomics Chromium single‐cell platform, we performed scRNA‐seq on the PBMCs of patients.^44^ Beads with unique molecular identifiers (UMIs) and cell barcodes were loaded into PBMCs in suspension in a nearly saturated state, so that each cell was paired with beads (Gel Bead‐in Emulsion). After exposure to the cell lysis buffer, the polyadenylated RNA molecules hybridized to the beads. The beads were then transferred into a single tube for reverse transcription. During cDNA synthesis, each cDNA molecule was labelled with UMI and a cell tag at the 5′ end of its source cell. To prepare a library, sequencing was used to randomly interrupt the whole transcriptome amplification product to enrich the 3′ end of the transcript connected to the cell barcode and UMI. All remaining procedures, including library construction, were conducted in accordance with standard manufacturing protocols. The sequencing library was quantified using a high‐sensitivity DNA chip (Agilent) and Qubit high‐sensitivity DNA analysis (Thermo Fisher Scientific) on bioanalyzer 2100. The library was sequenced on NovaSeq6000 (Illumina) with the 2 × 150 chemical method.

### 4.4 Data dimensionality reduction clustering, cell grouping and differentially expressed gene analysis

Unsupervised clustering and differential gene analyses were performed using the Seurat package.^45^ Reads were processed using the Cell Ranger 2.1.0 pipeline with default and recommended parameters. FASTQs generated from the Illumina sequencing output were aligned to the human genome, version GRCm38, by using the STAR algorithm.^46^ Next, gene‐barcode matrices were generated for each individual sample by counting UMIs and filtering noncell‐associated barcodes. Finally, we generated a gene‐barcode matrix containing barcoded cells and gene expression counts. This output was then imported into the Seurat (v2.3.0) R toolkit for quality control and downstream analysis of our scRNA‐seq data.^45^ After the data were normalized (“NormalizeData” function in the Seurat software package), variable gene subsets were extracted. When a strong relationship was shown between variability and average gene expression, the variable gene was determined. Next, “findinintegrationanchors” and “integrateddata” in the Seurat package were used to identify the “anchors” between data sets, and the data from different samples were integrated.^45^, ^47^ Principal component analysis was performed, and after data were scaled, the data dimension was reduced to the first 30 principal components. The t‐distributed stochastic neighbourhood embedding (t‐SNE) method was used to achieve visual clustering on a two‐dimensional map.^48^ And the cell‐type identification was performed according to the data sets published by 10x genomics (https://www.10xgenomics.com/resources/datasets/10‐k‐human‐pbm‐cs‐multiplexed‐2‐cm‐os‐3‐1‐standard). The cell ratio was calculated according to the real number of cells obtained in each cluster with R.

We actually plotted the fraction of cells in a cluster at each time point and the numbers combining all the cells in these clusters from all patients.

### 4.5 Hallmark gene sets analysis

According to “hallmark” gene sets refined and made concise by Liberzon et al. from the Molecular Signatures Database, hall mark gene sets analysis was conducted.^49^

### 4.6 Pathway analysis

The GSEA function of the R package clusterProfiler^50^ was used to identify a set of a priori defined genes that showed statistically significant differences between the given clusters.

### 4.7 Cell–cell communication analysis

CellphoneDB^51^ was used for the systematic analysis of cell–cell communication molecules. Its accuracy relies on public resources to annotate receptors and ligands.

## CONFLICTS OF INTEREST

The authors declare no conflicts of interest.

## Supporting information



Supporting Information

Supporting Information

Supporting Information

Supporting Information

Supporting Information

Supporting Information

Supporting Information

Supporting Information

Supporting Information

Supporting Information

Supporting Information

Supporting Information

Supporting Information

Supporting Information

Supporting Information

Supporting Information

Supporting Information

Supporting Information

Supporting Information
