# Supplementary figures and images for: An atlas of dynamic peripheral blood mononuclear cell landscapes in human perioperative anaesthesia/surgery
Source: Clin Transl Med. 2022 Jan 21;12(1):e663. doi: 10.1002/ctm2.663 (PMC8782495; doi:10.1002/ctm2.663)

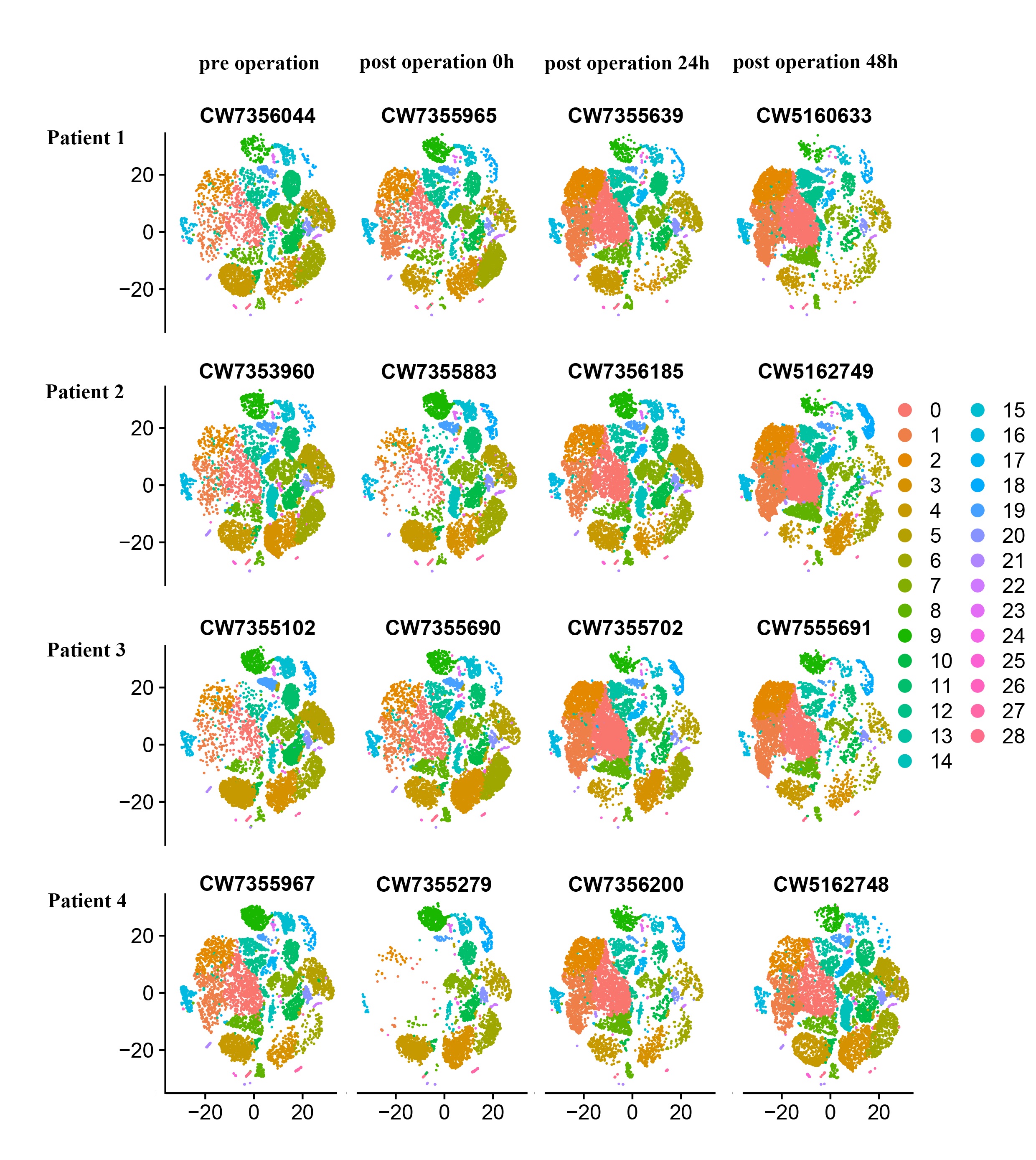

Supplement: Supplementary file 1 — Supporting Information [file CTM2-12-e663-s019.jpg]

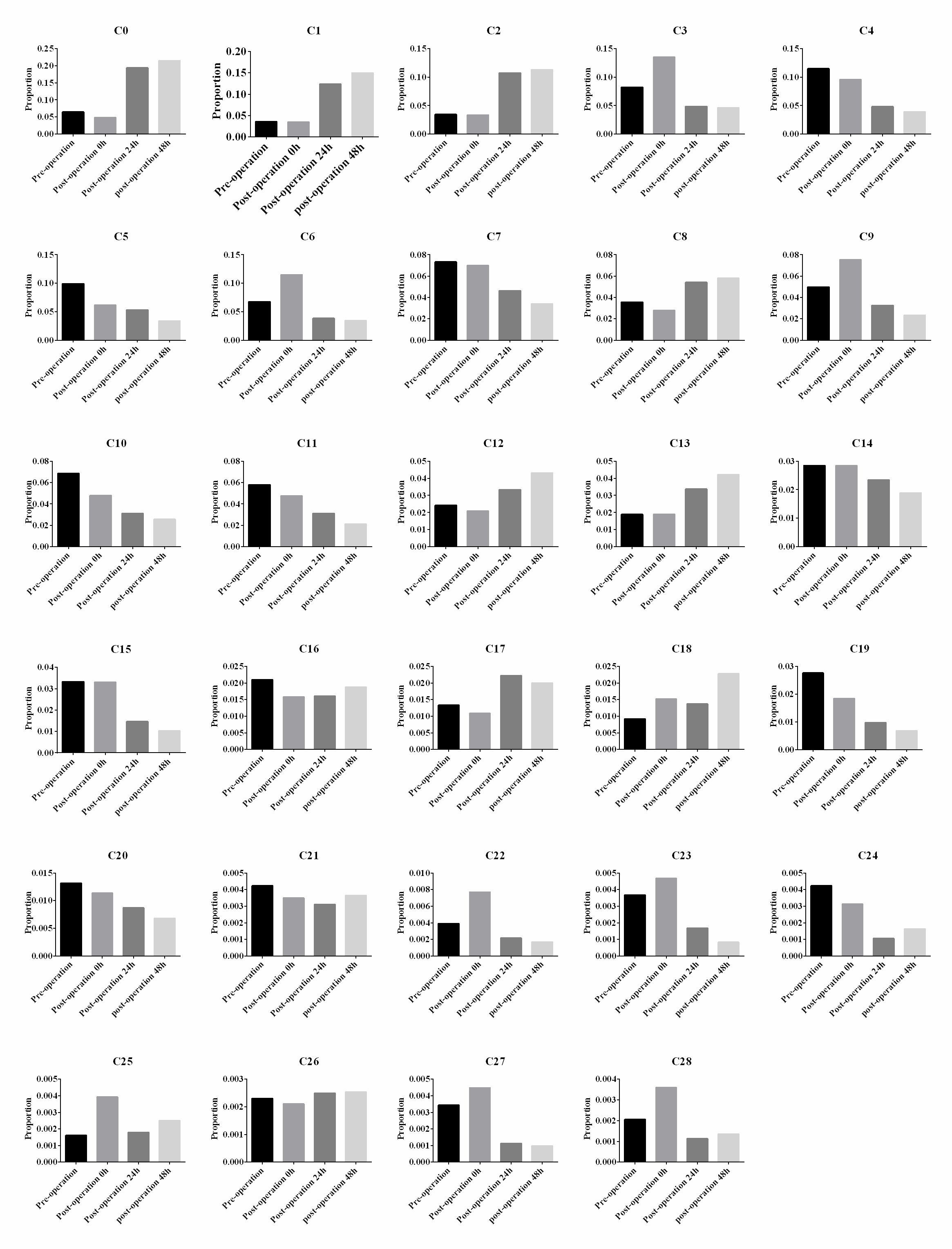

Supplement: Supplementary file 2 — Supporting Information [file CTM2-12-e663-s002.jpg]

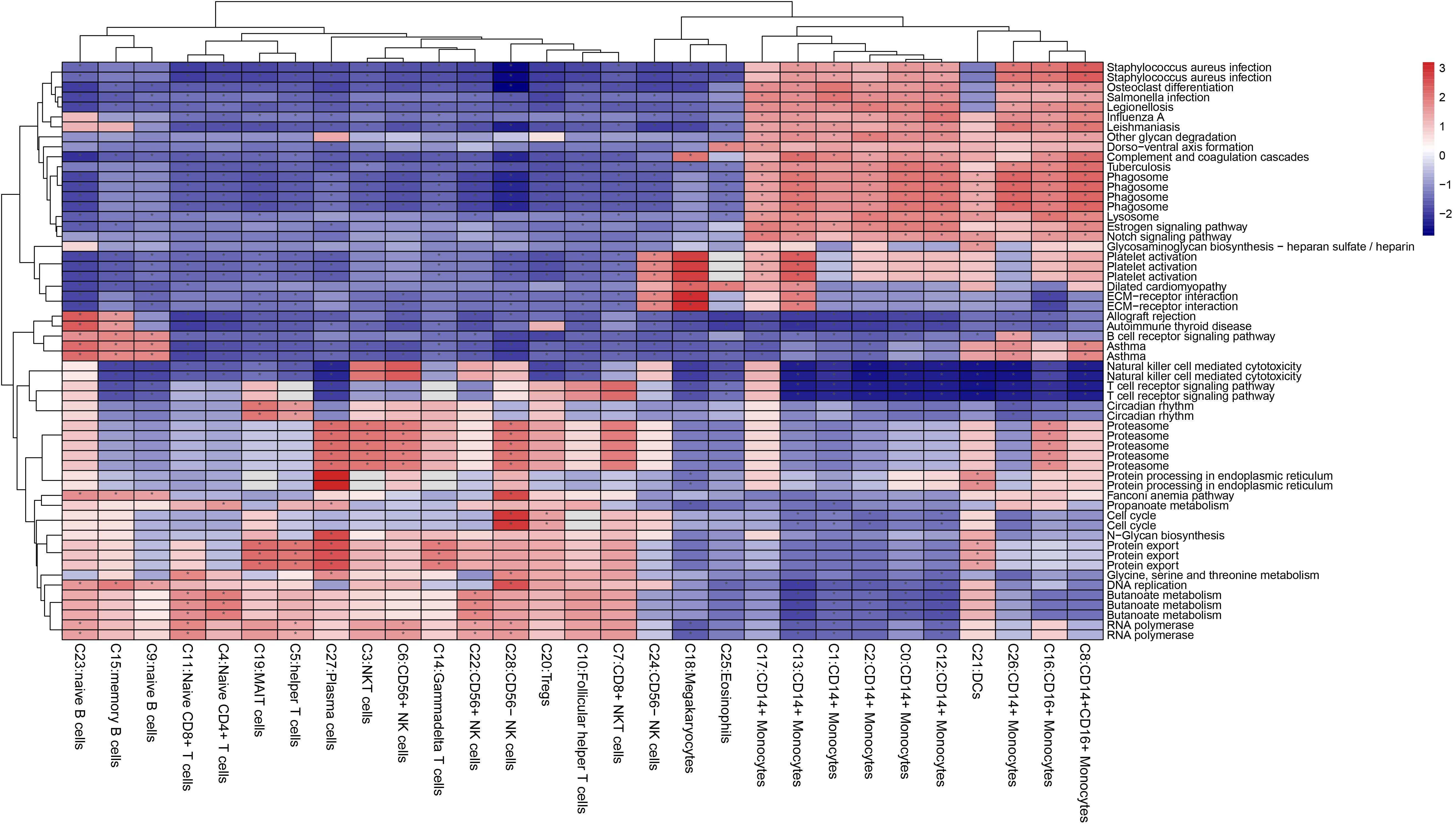

Supplement: Supplementary file 3 — Supporting Information [file CTM2-12-e663-s010.jpg]

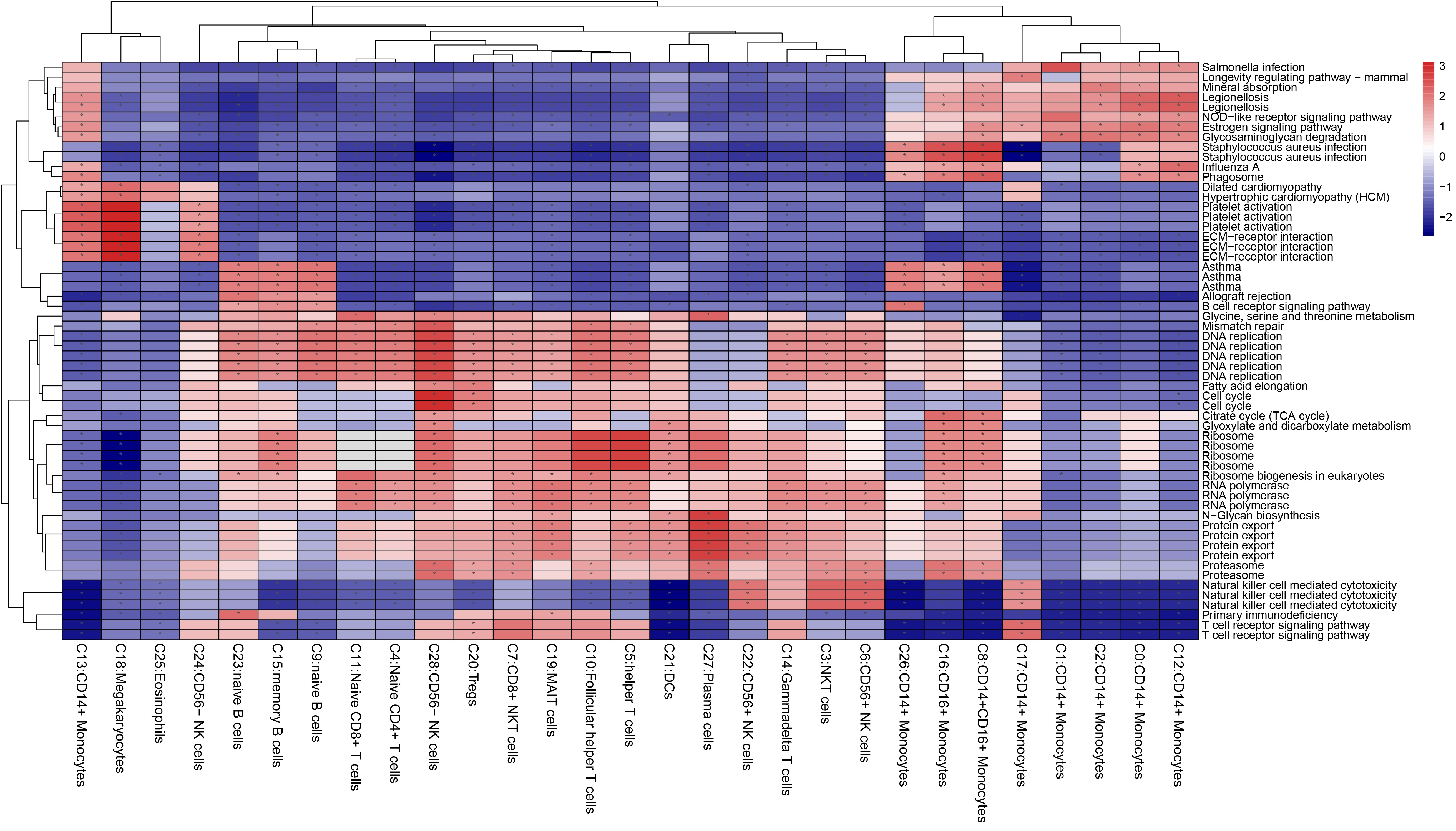

Supplement: Supplementary file 4 — Supporting Information [file CTM2-12-e663-s004.jpg]

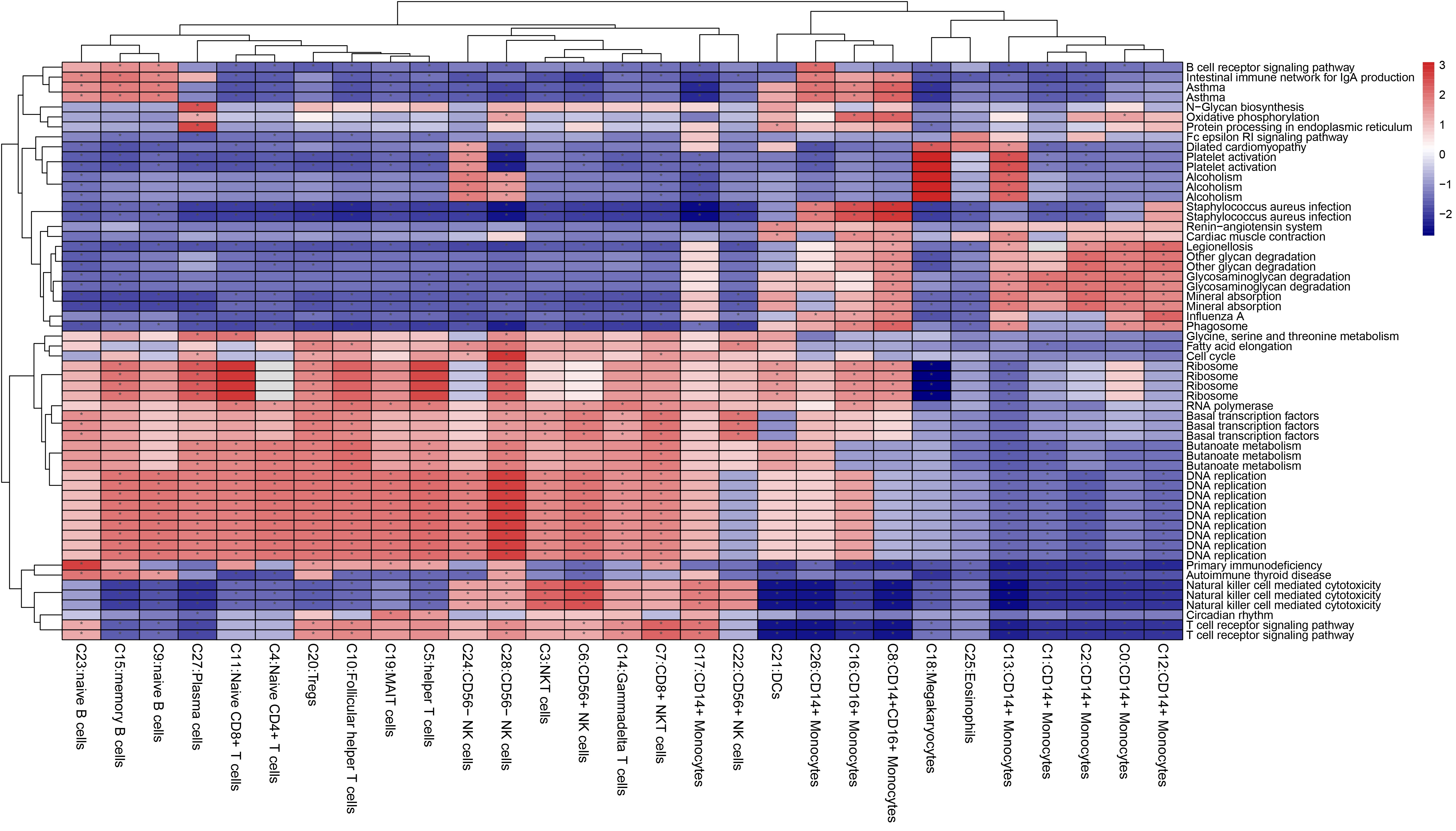

Supplement: Supplementary file 5 — Supporting Information [file CTM2-12-e663-s014.jpg]

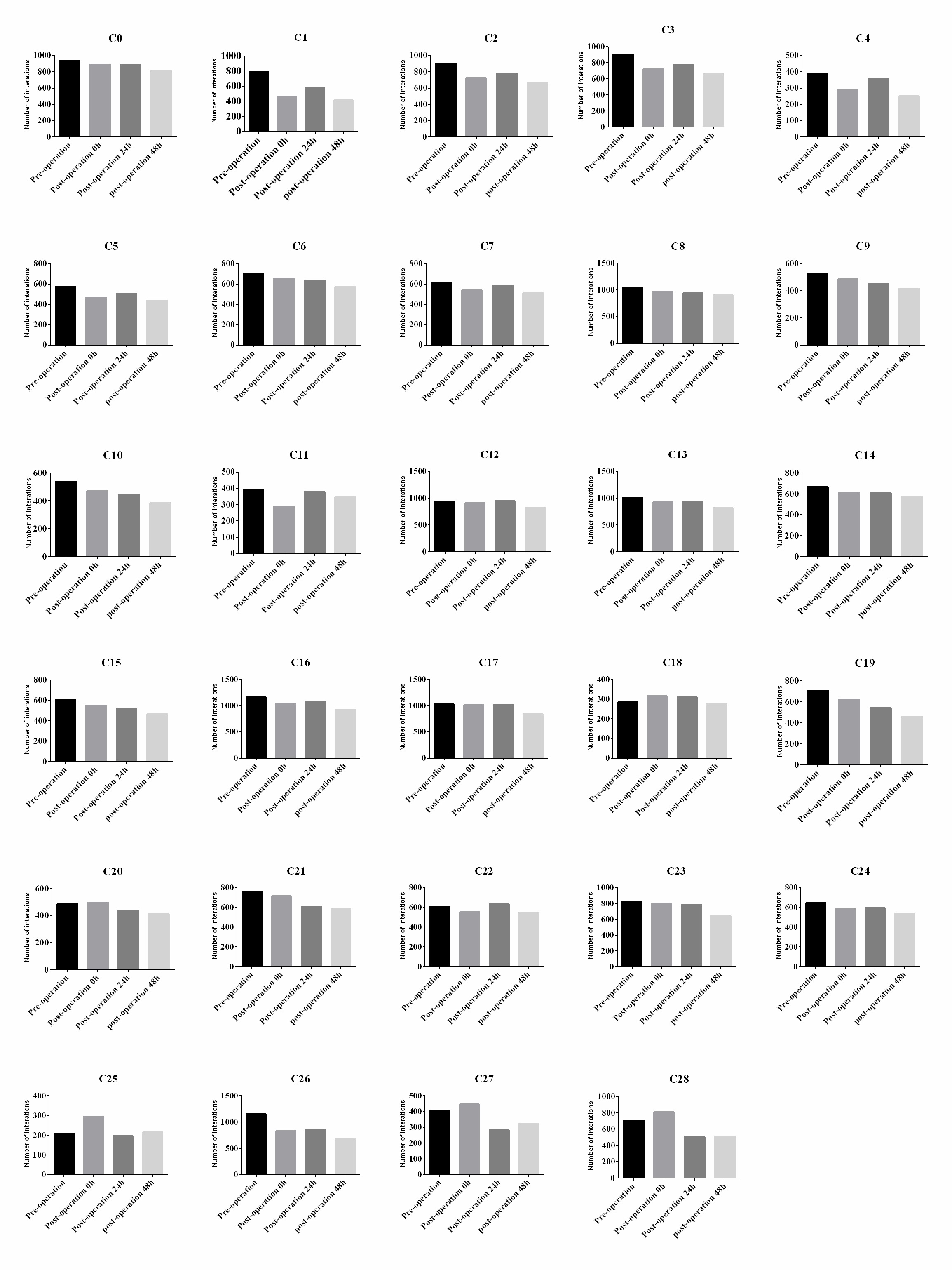

Supplement: Supplementary file 6 — Supporting Information [file CTM2-12-e663-s015.jpg]

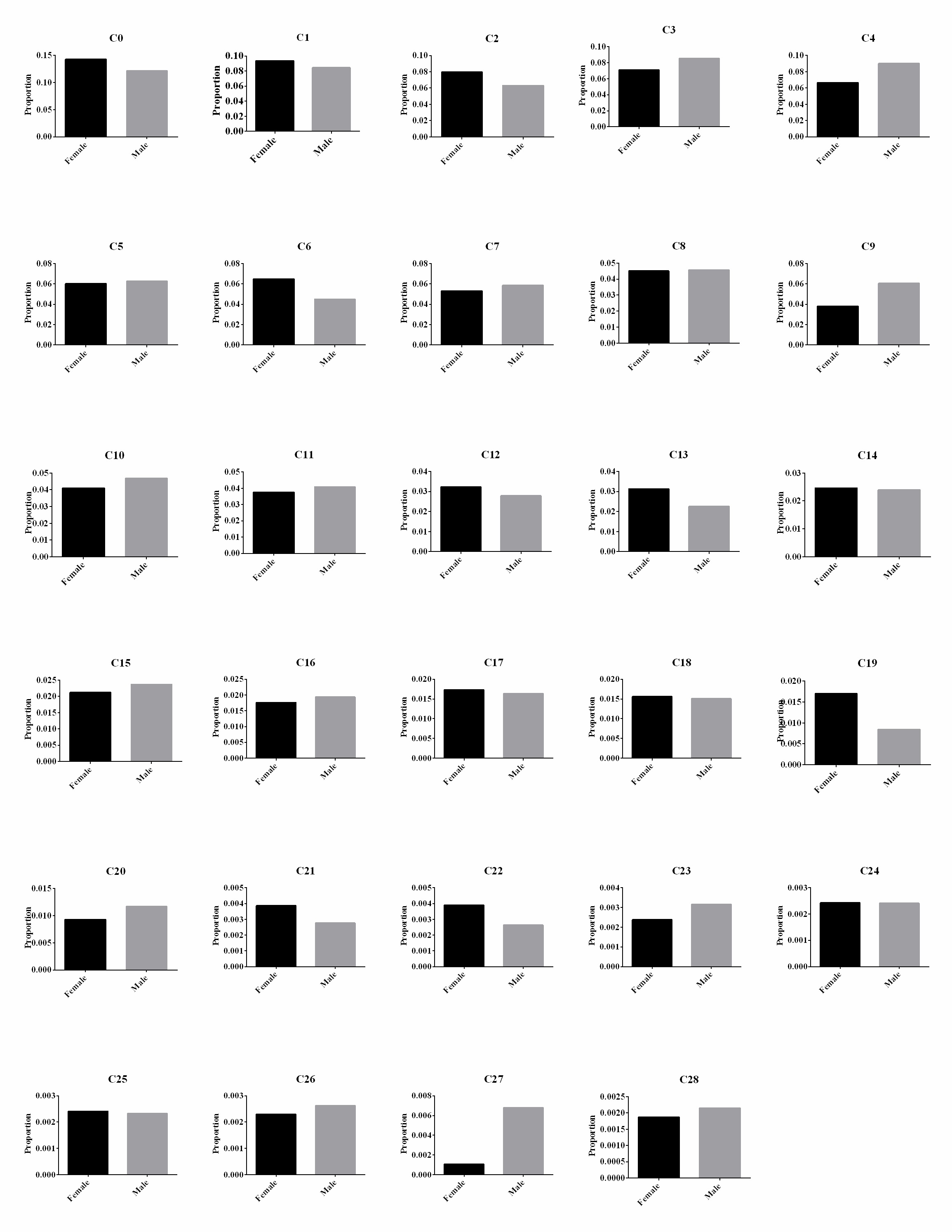

Supplement: Supplementary file 7 — Supporting Information [file CTM2-12-e663-s008.jpg]

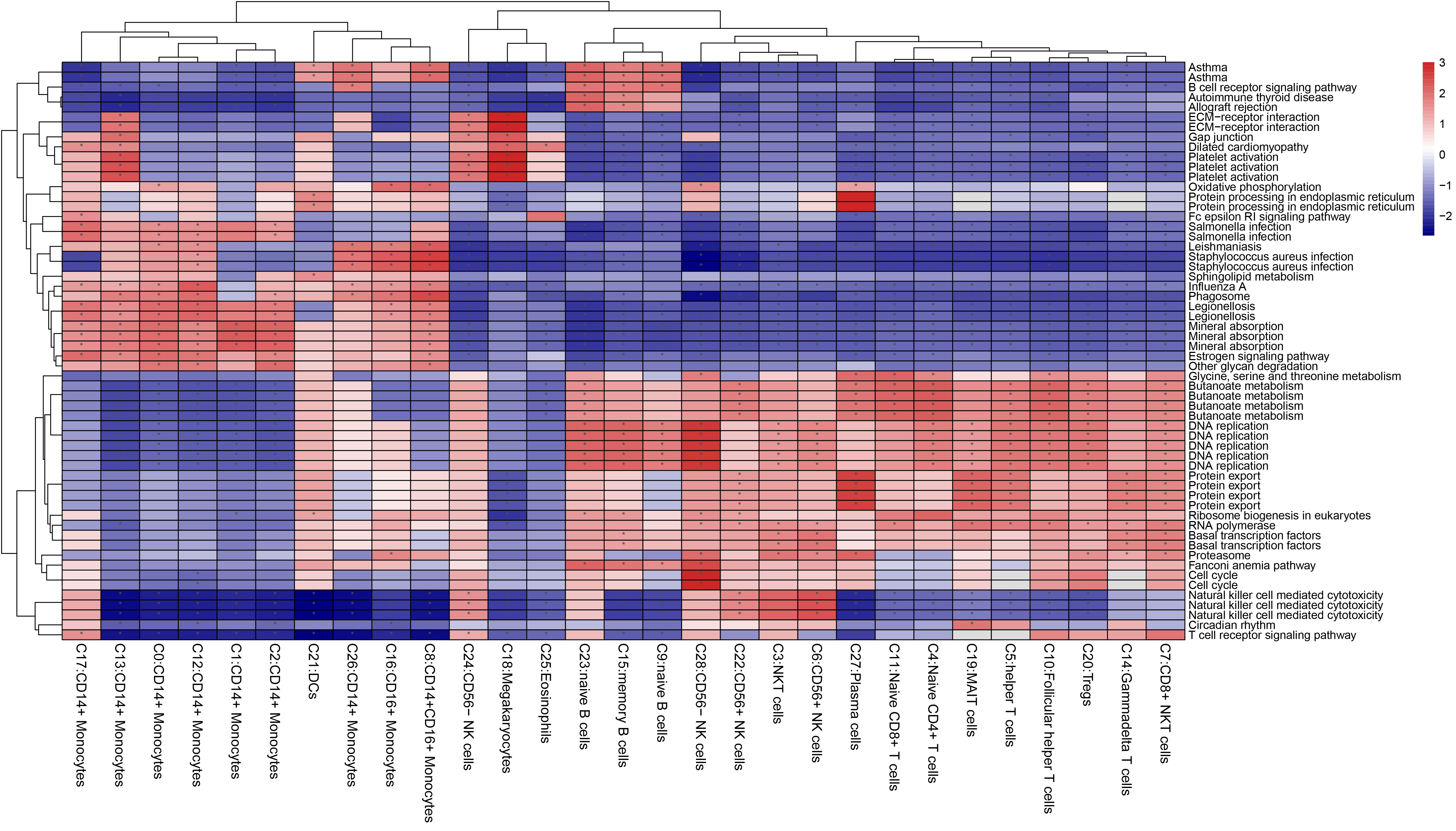

Supplement: Supplementary file 8 — Supporting Information [file CTM2-12-e663-s007.jpg]

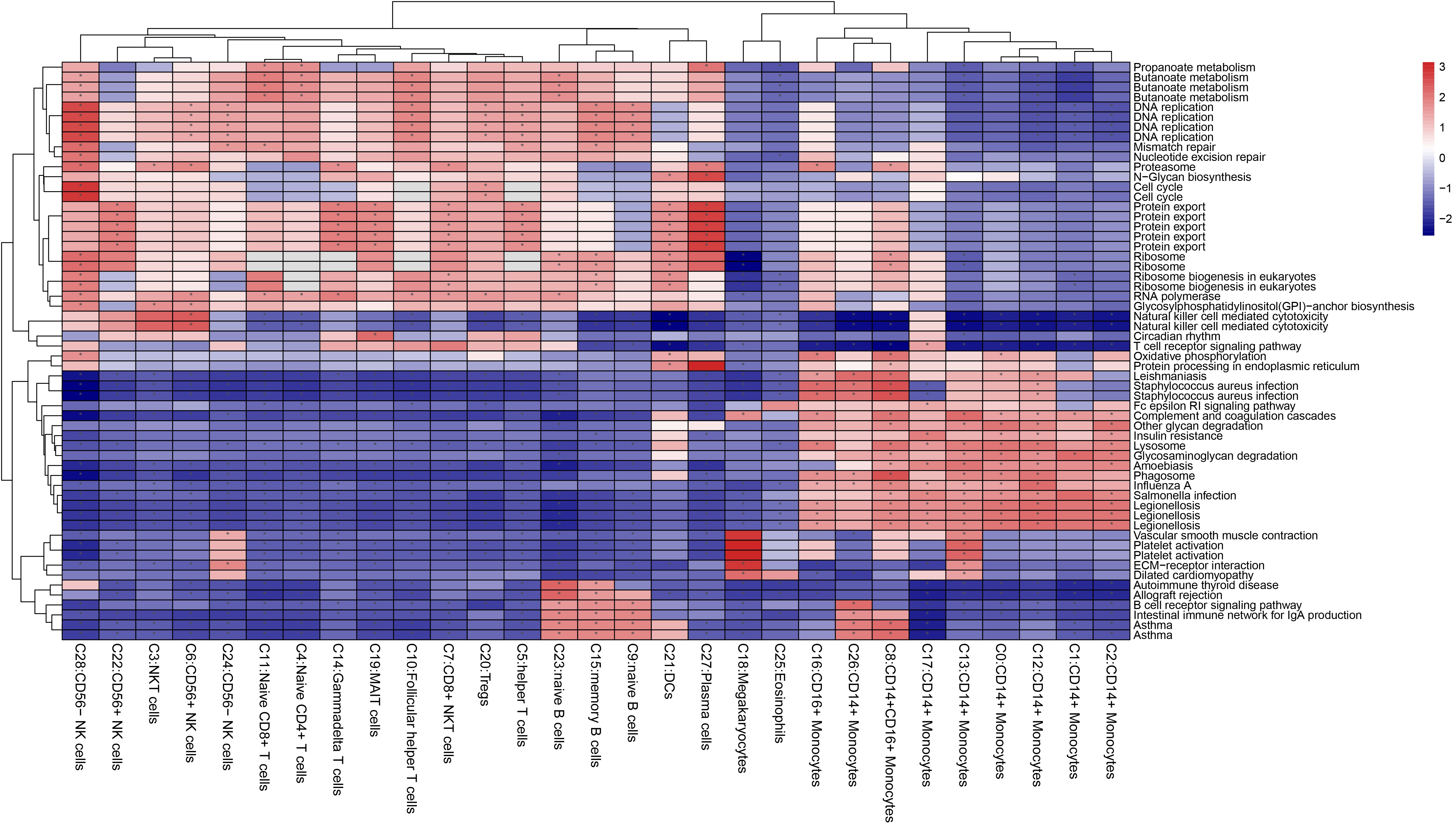

Supplement: Supplementary file 9 — Supporting Information [file CTM2-12-e663-s017.jpg]

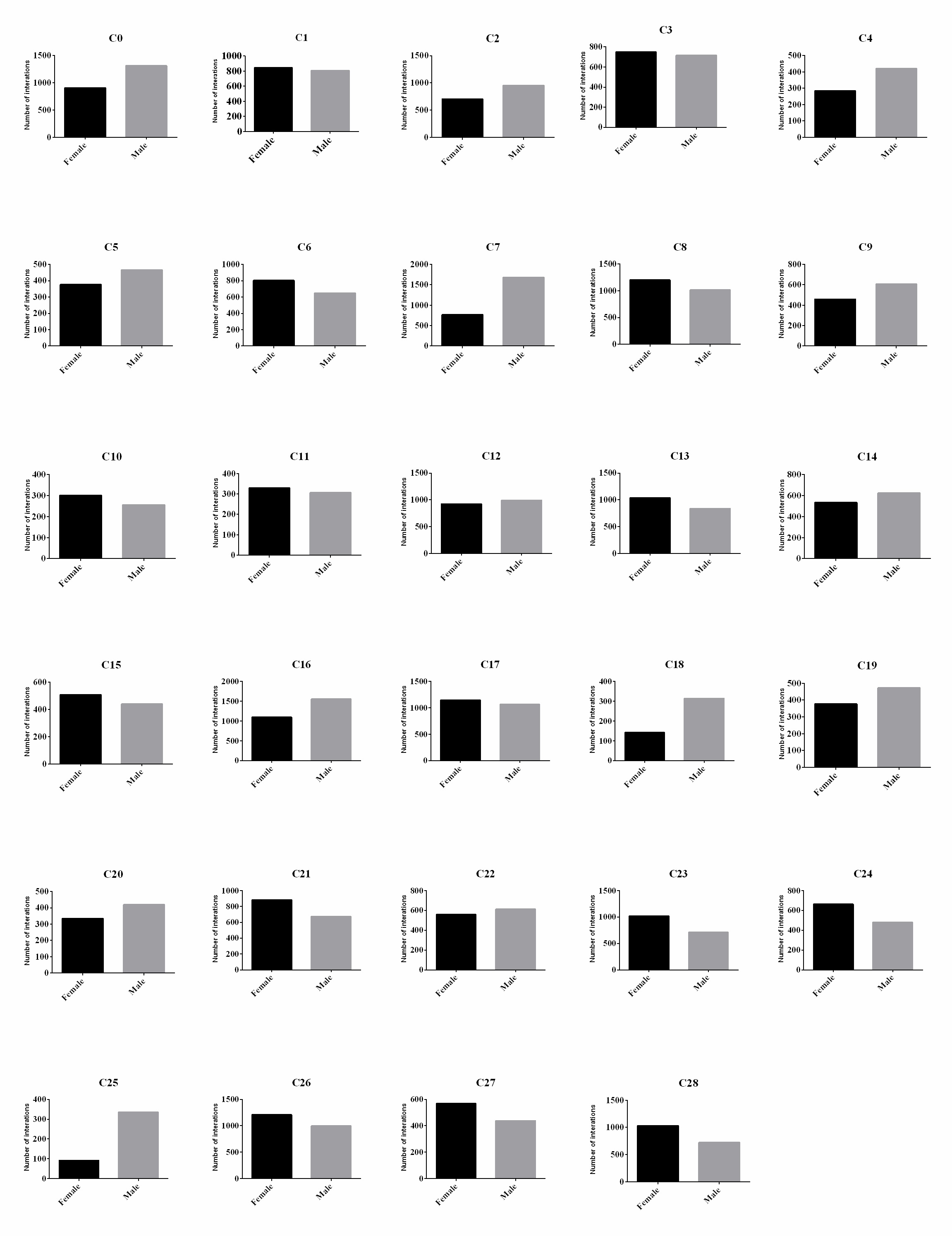

Supplement: Supplementary file 10 — Supporting Information [file CTM2-12-e663-s016.jpg]

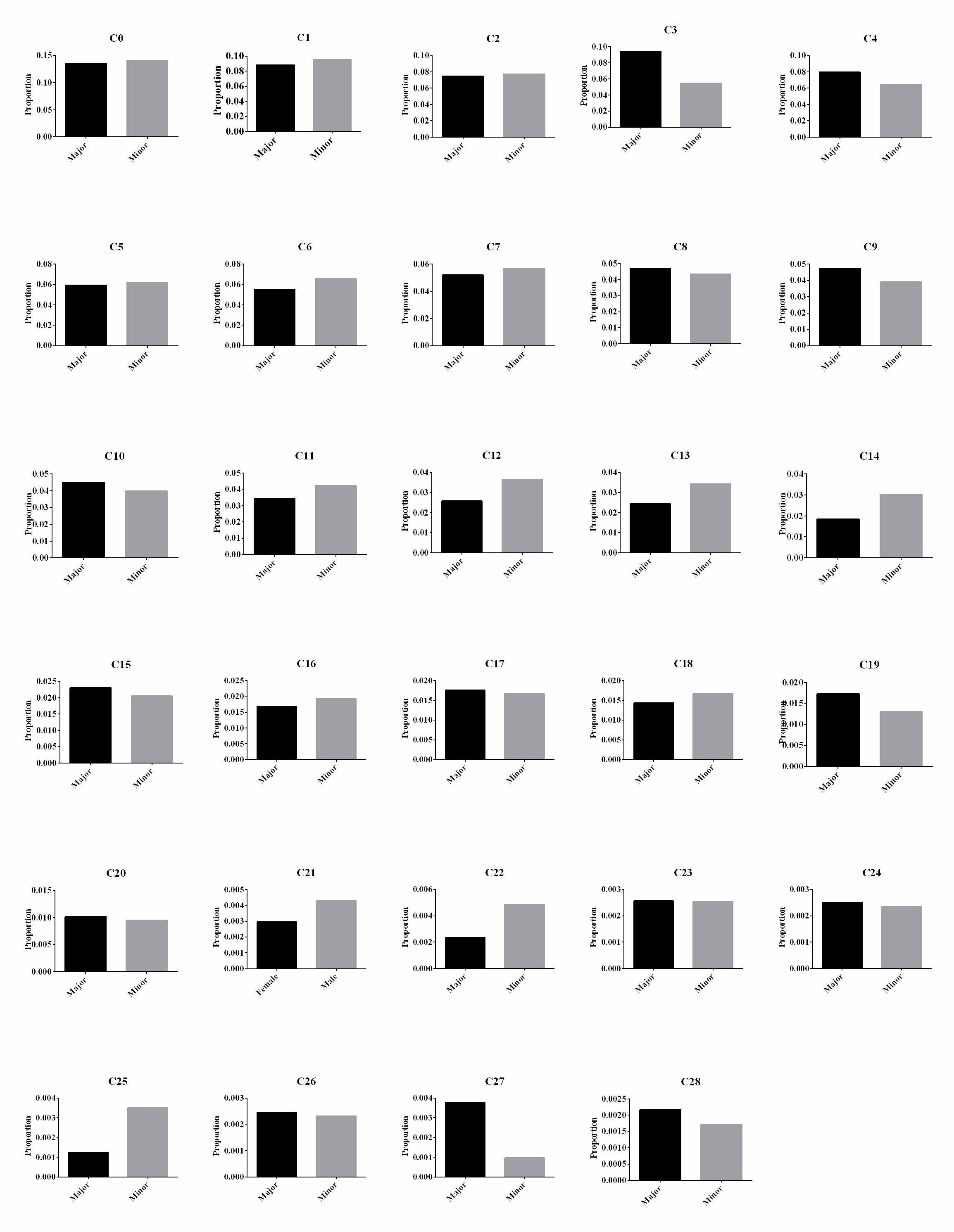

Supplement: Supplementary file 11 — Supporting Information [file CTM2-12-e663-s005.jpg]

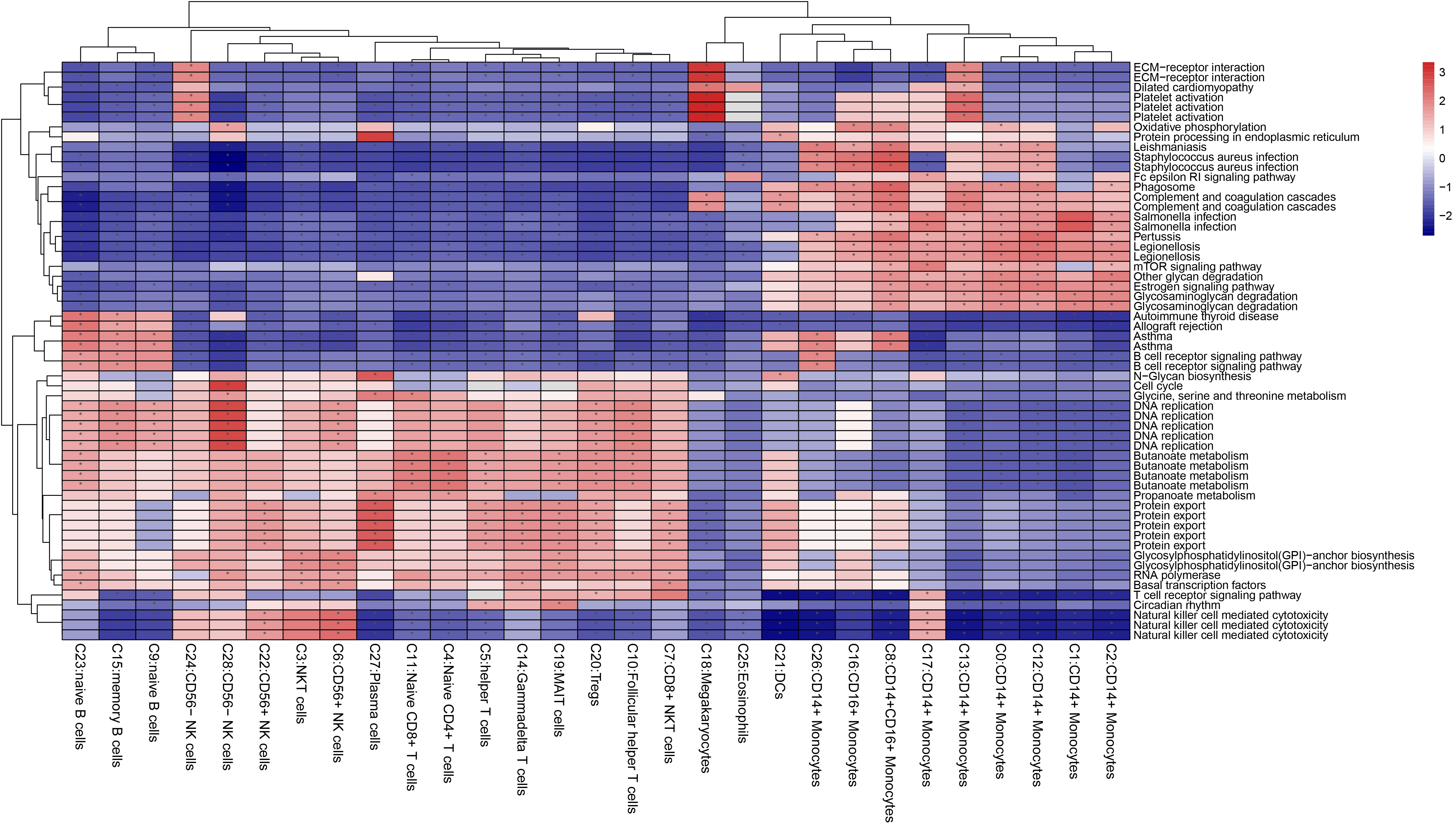

Supplement: Supplementary file 12 — Supporting Information [file CTM2-12-e663-s001.jpg]

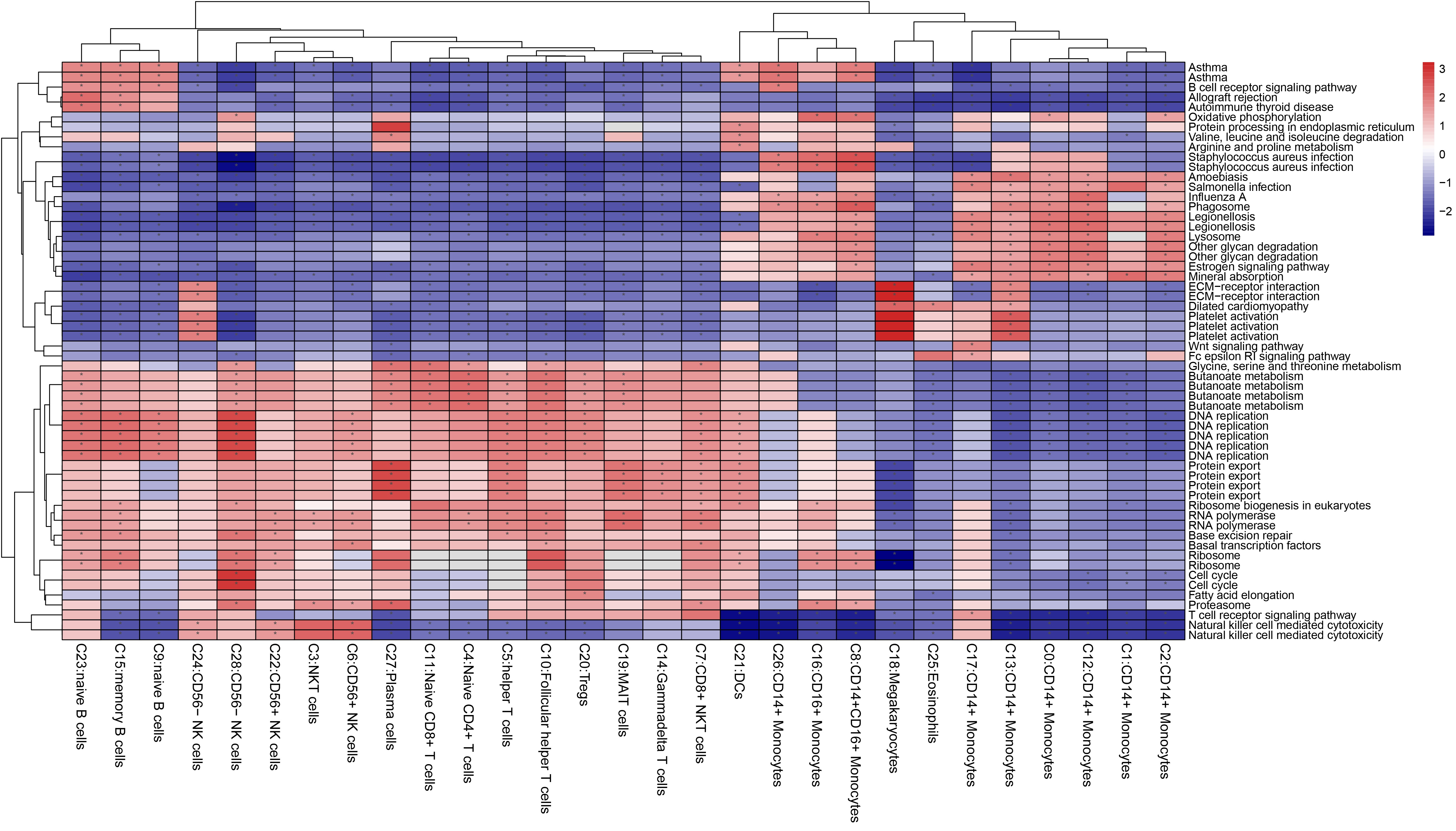

Supplement: Supplementary file 13 — Supporting Information [file CTM2-12-e663-s011.jpg]

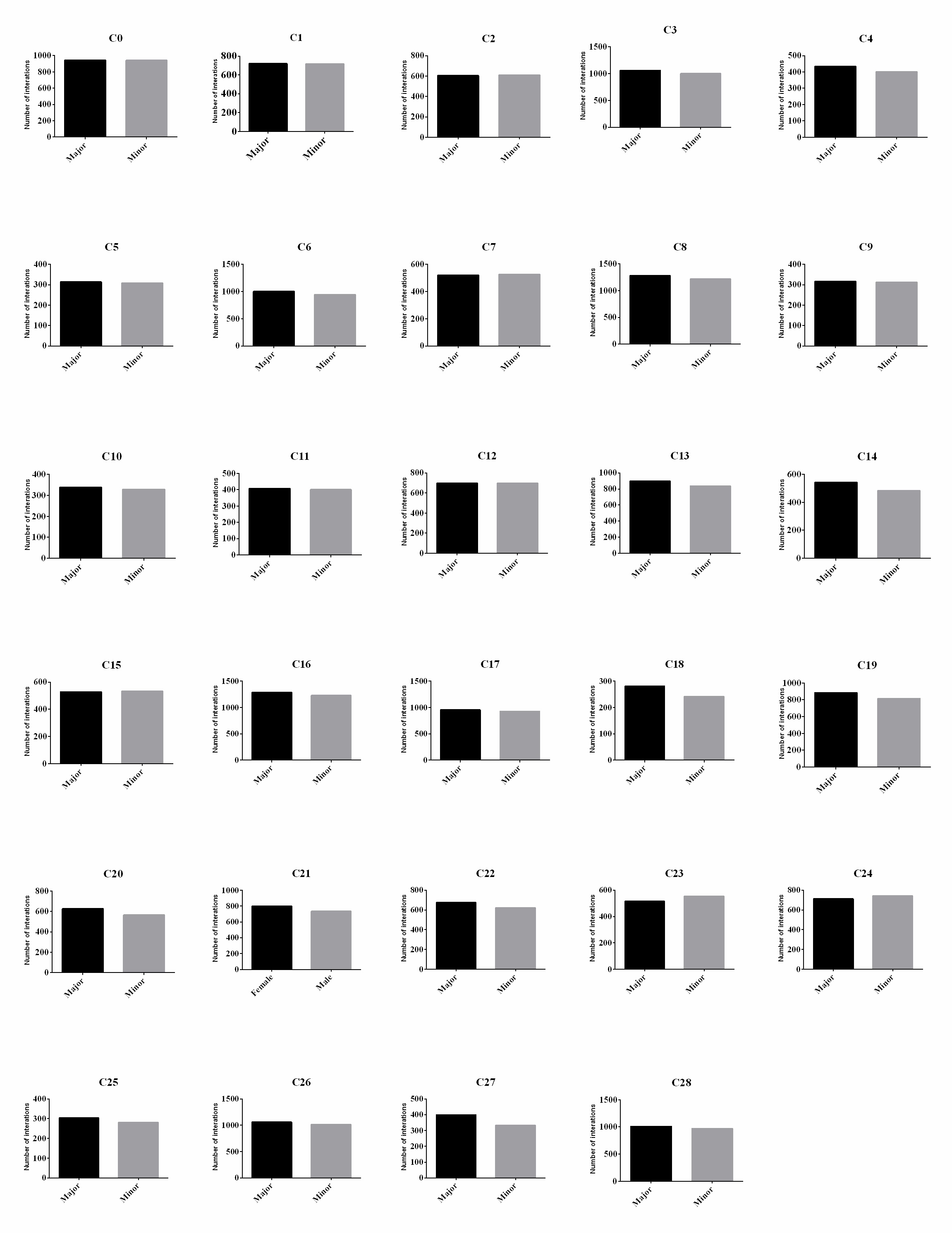

Supplement: Supplementary file 14 — Supporting Information [file CTM2-12-e663-s018.jpg]

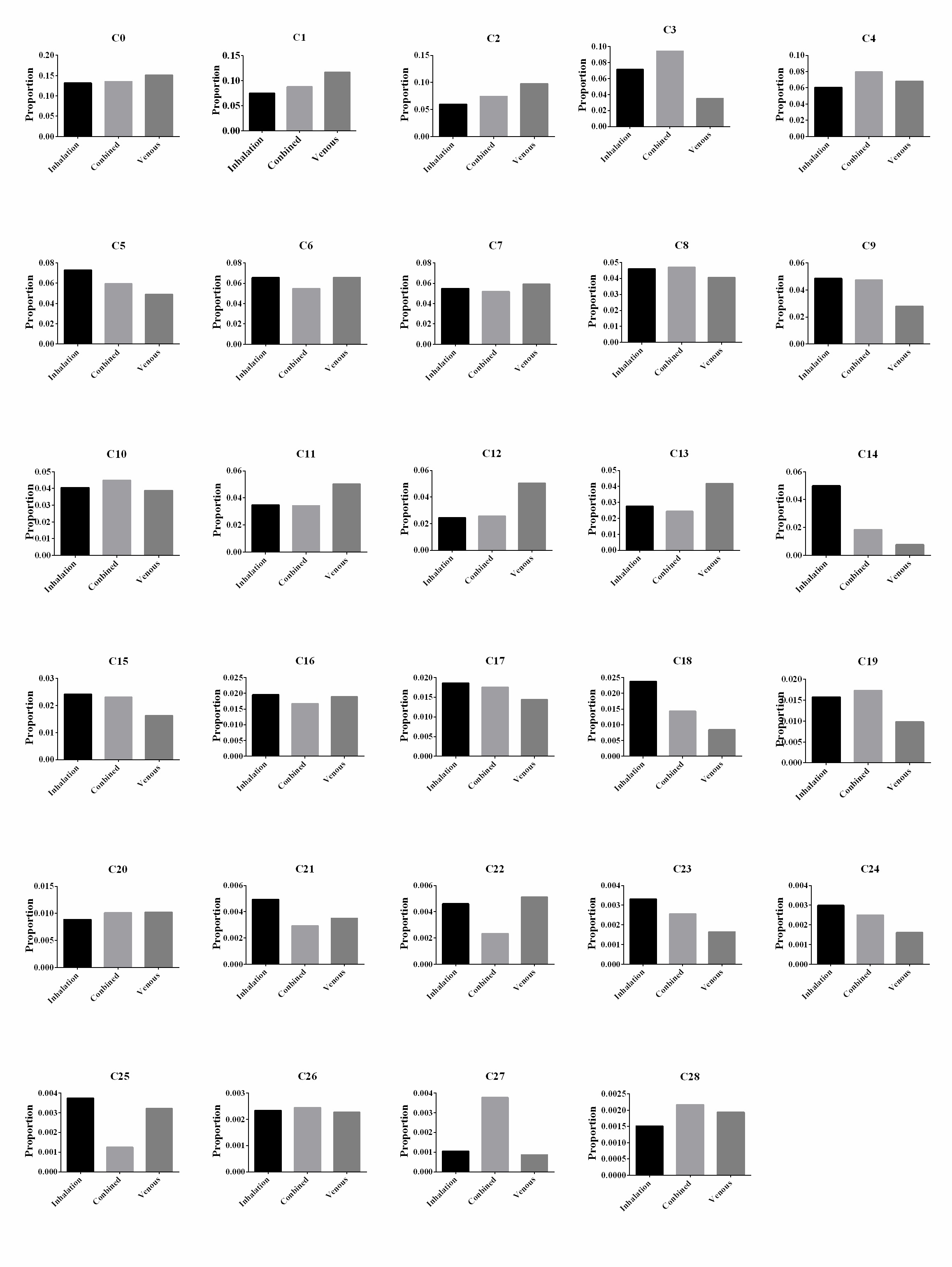

Supplement: Supplementary file 15 — Supporting Information [file CTM2-12-e663-s003.jpg]

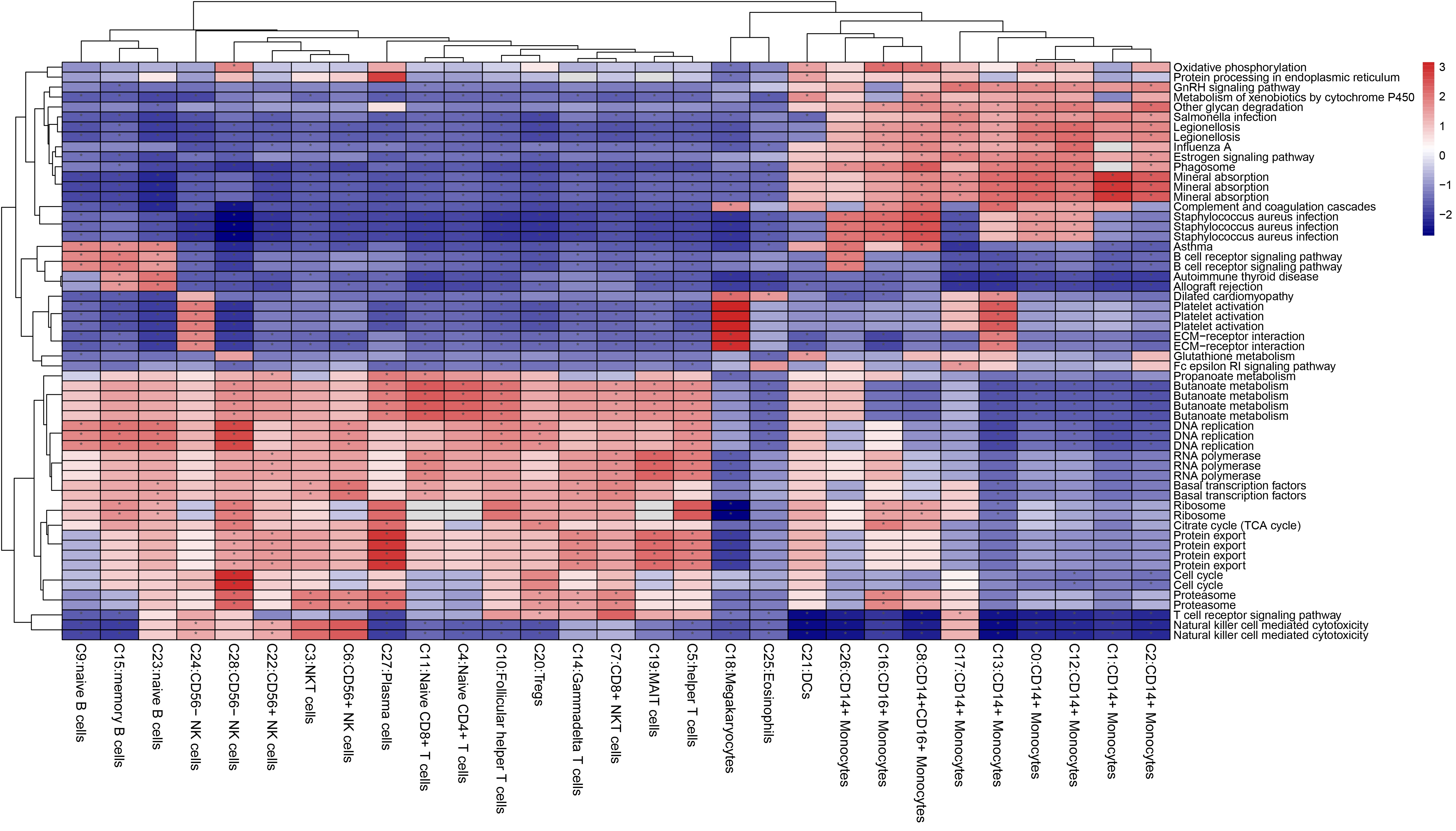

Supplement: Supplementary file 16 — Supporting Information [file CTM2-12-e663-s012.jpg]

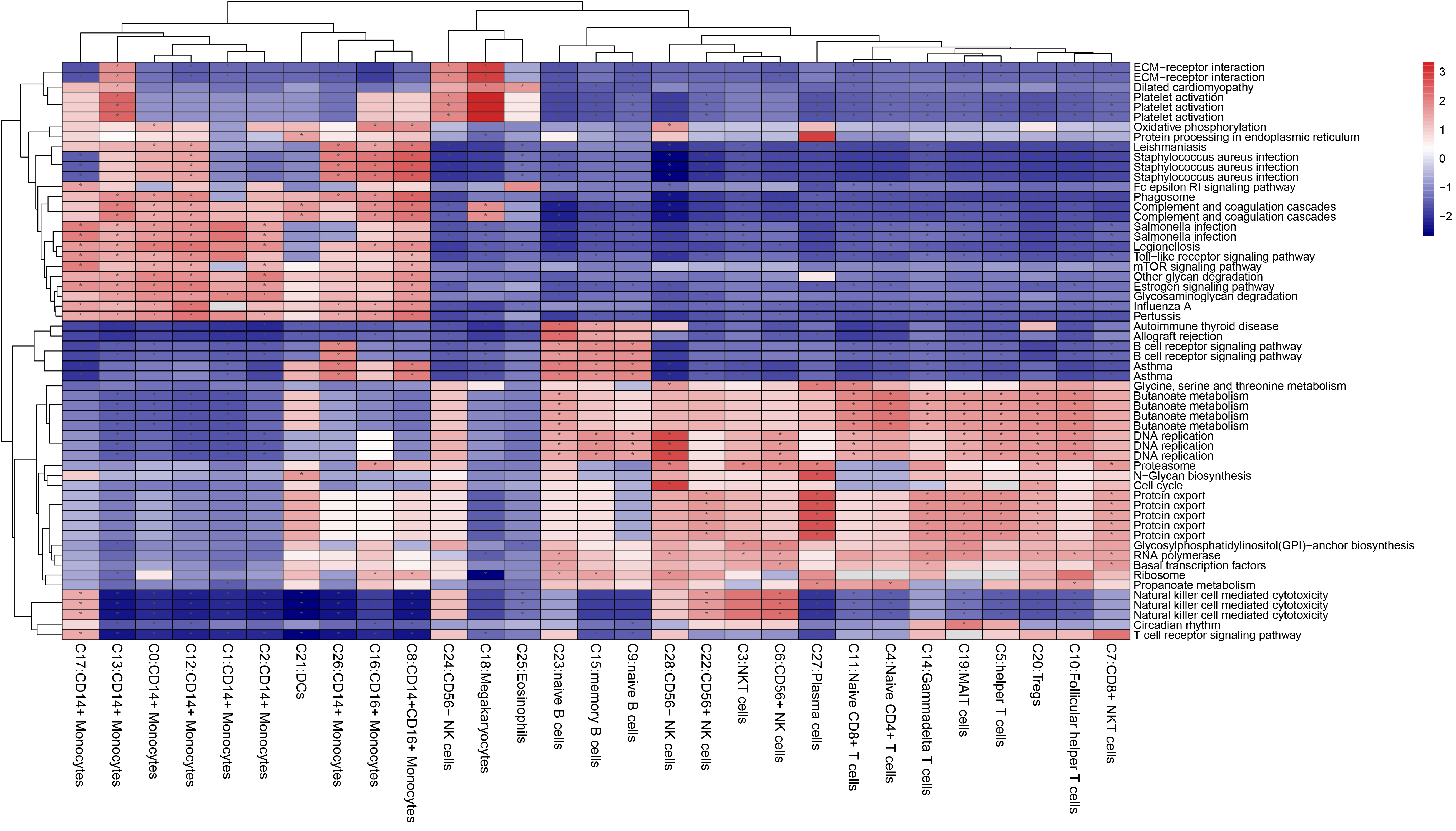

Supplement: Supplementary file 17 — Supporting Information [file CTM2-12-e663-s013.jpg]

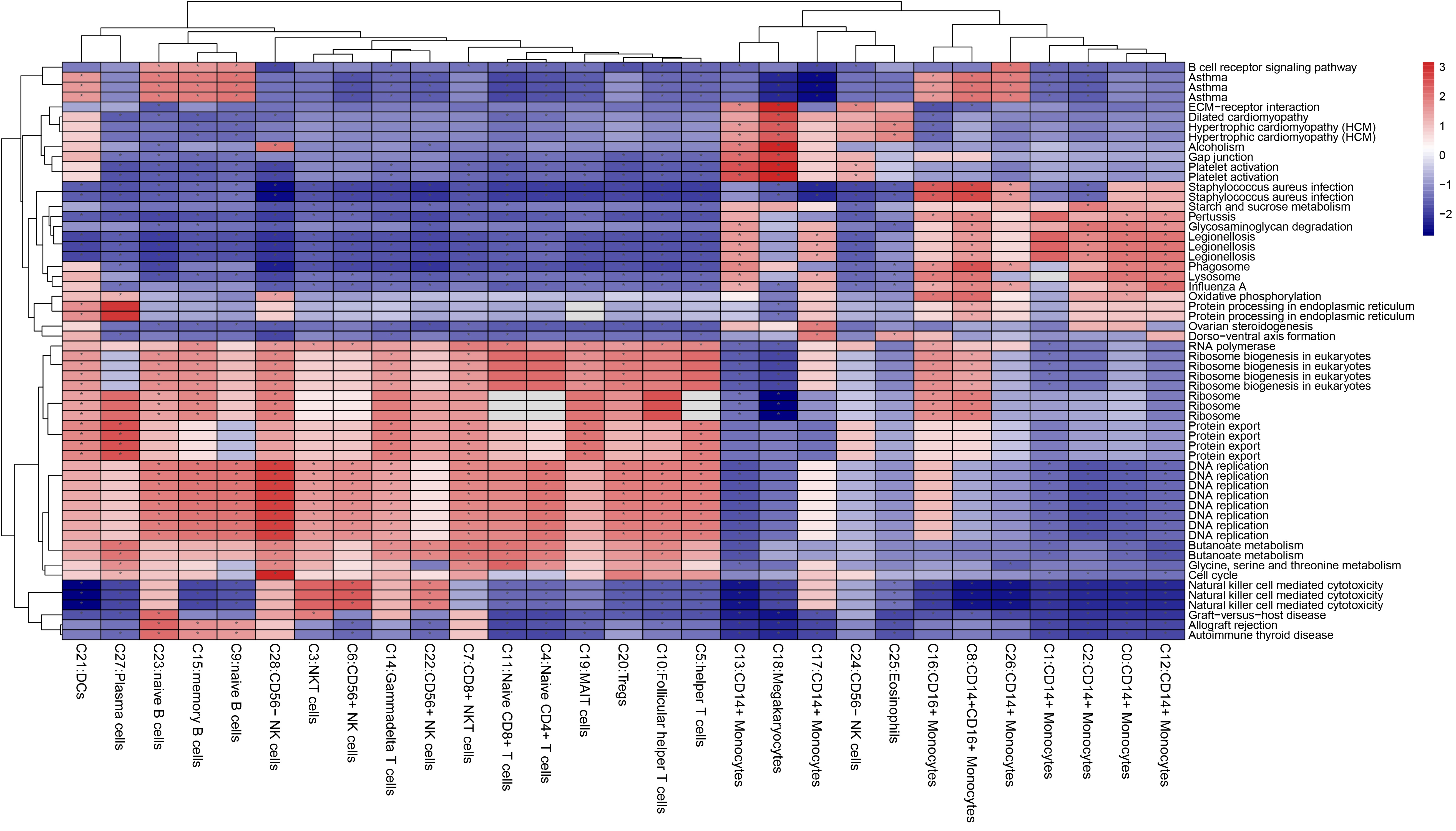

Supplement: Supplementary file 18 — Supporting Information [file CTM2-12-e663-s009.jpg]

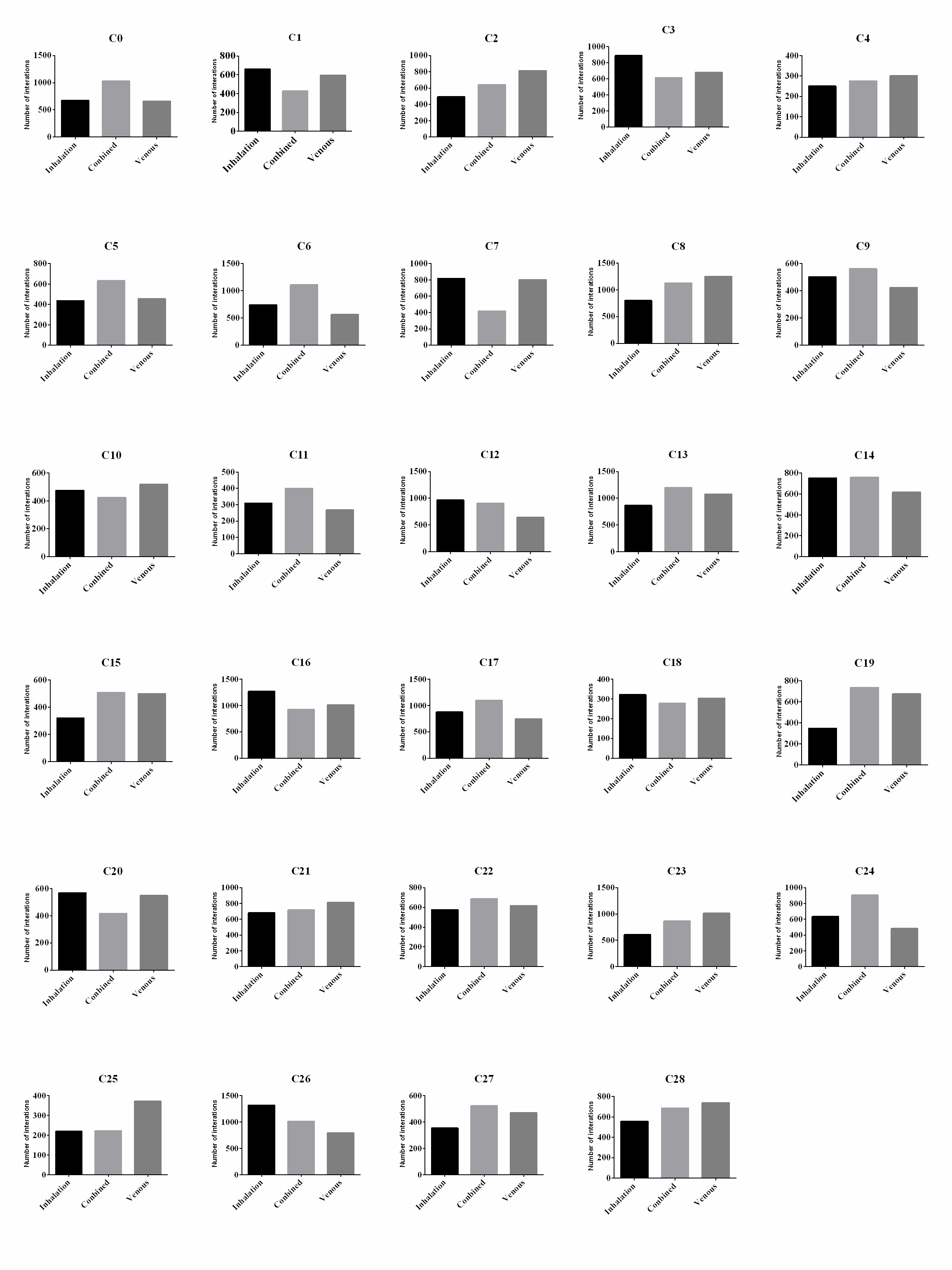

Supplement: Supplementary file 19 — Supporting Information [file CTM2-12-e663-s006.jpg]
